# Sleep quality among workers in the health sector exposed to the COVID-19 pandemic

**DOI:** 10.1371/journal.pone.0268933

**Published:** 2022-12-01

**Authors:** Adelina Tmava-Berisha, Frederike T. Fellendorf, Michaela Ratzenhofer, Alexander Maget, Martina Platzer, Susanne A. Bengesser, Armin Birner, Robert Queissner, Elena Schönthaler, Nina Dalkner, Melanie Lenger, Eva Z. Reininghaus

**Affiliations:** Department of Psychiatry and Psychotherapeutic Medicine, Medical University of Graz, Graz, Austria; University of Palermo, ITALY

## Abstract

The ongoing pandemic of coronavirus disease (COVID-19) is a global health crisis that has posed enormous pressure on workers in the health sector (WHS), having a massive impact on their mental health. In this study, we aimed to evaluate the sleep quality of WHS during the pandemic and compare frontline WHS to those who are not directly engaged in the care of COVID-19 patients. This cross-sectional, self-reported online survey assessed the sleep quality of WHS in Austria using the Pittsburgh Sleep Quality Index (PSQI). The same questionnaire was sent out two times. Due to the unequal sample and anonymity of the study participants, we analyzed the data of each time point separate from each other. The first study was conducted in April/May 2020, during the first lockdown in Austria (Study1), and the second study was conducted in July/August 2020, when the social restrictions were loosened (Study2). T-test was used to compare the mean values of PSQI scores between frontline vs. non-frontline WHS, while two two-way ANCOVAs were used to analyze differences in the PSQI mean scores (controlled for age) for male vs. female between frontline vs. non-frontline WHS. During the first lockdown in Austria (Study1) we identified a shorter sleep duration of frontline WHS compared to the non-frontline group, however the difference in global PSQI score between these groups was statistically not significant. In the period after loosened restrictions (Study2) the sleep quality, sleep latency, sleep duration, sleep efficiency and global PSQI score was worse in frontline WHS compared to the non-frontline WHS. Furthermore, female WHS scored higher in the PSQI indicating a worse sleep than male WHS. In addition, nurses and nursing assistants had a higher prevalence of poor sleep quality than other occupational groups. Our results indicate that the COVID-19 pandemic negatively impacts the sleep of WHS, affecting particularly frontline WHS. Preventive interventions aiming to promote good sleep quality in WHS during a healthcare crisis like this pandemic are essential to enhance resilience and mitigate the vulnerability of this specific population.

## Introduction

The global ongoing coronavirus disease (COVID-19) pandemic is caused by the severe acute respiratory syndrome coronavirus 2 (SARS-CoV-2). Up to now, COVID-19 has affected 223 countries and territories worldwide, thus becoming a major global health concern [1].

To contain the pandemic’s spread, governments worldwide have passed new laws and implemented restrictions on private and public issues. Although, to date, the United States Food and Drug Administration has approved drug treatments for COVID-19 and has authorized others for emergency use during this public health emergency, the most effective weapon that society has against this virus is the prevention of its spread [2]. Hand hygiene, social distancing, and quarantine are essential to combat the virus [3]. Also, the use of personal protective equipment, including face protecting masks, has ensured greater control of the spread of the virus [4]. Furthermore, the large-scale use of vaccines has favorably changed the initial scenario of the pandemic by reducing contagions and drastically decreasing the hospitalization of people infected with the virus. Among the organizational measures checking the regularity of vaccination or regular COVID- testing, the constant cleaning of workplaces with the indicated detergents, and use of systems to improve the natural exchange of the air are proved to be very effective in decreasing the possible presence of the virus in workplaces [4].

In Austria, on March 15th, 2020, the government announced a nationwide lockdown, enforced exit restriction, social distancing, and wearing a face mask in public [5]. The country’s executive eased the first restrictions on April 13th, where small shops reopened, followed by the reopening of restaurants and schools by the end of May 2020.

The rapid increase in the number of patients infected with the virus has placed extraordinary healthcare systems demands worldwide. Medical Staff had to work under enormous care pressure dealing with longer duty hours, work in isolation units, and uncertainty regarding effective disease control [6]. Furthermore, worries about contagion, their families’ health, and colleagues’ safety at the workplace tormented healthcare professionals. Lockdown and social life limitations have also contributed to widespread stress and promoted mental health problems [7–9].

It is already known that stress can have consequences on biological systems as well as different aspects of private and work-related life [10]. Neuroendocrine factors, immunological aspects as well as genetic and epigenetic factors are involved. In another study greater individual reactivity to stress predicted higher risk of dementia while measures of work-related stress (job dissatisfaction and high job demands) were not associated with dementia risk [11].

Night shift work seems to play a role in generating stress in the workers. Due to a study among security guards, night-time shift cortisol levels significantly increase before and after the work shifts. According to that, cortisol and blood pressure can be considered sensitive markers of biological responses to severe work stress [12]. In addition, research analyses among nurses working in a shift system associated sleep disturbances with night work and professional burnout [13].

The evolution of the COVID-19 pandemic has forced multiple abrupt adjustment demands in work with homeworking at the beginning of the pandemic and then return to work, mainly due to vaccination of the population. A long prospective study investigated the effect of adaptation demands on an Italian banking group in the job-related framework. Their data showed that massive adjustment demands in work and family routines represented a significant source of stress for workers, regardless of the different pandemic stages [14]. These results shed light on the need for a road map to promote workers’ gradual and structured adjustment.

Research on the association between working stress and sleep quality has been limited, despite the growing recognition of the consequences of sleep problems. A relationship between sleep problems and myocardial infarct has been demonstrated [15]. Poor sleep quality has also been linked to diabetes and hypertension, muscle pain, headaches, and gastrointestinal problems [16,17]. In addition, sleep disorders are a risk factor for mental health problems such as depression [18]. There are also public health consequences of sleep problems experienced by workers. A correlation between poor quality sleep and accidents, including motor vehicle accidents and workplace incidents, has been found [19]. Moreover, sleep disorders are associated with lower job performance, greater absenteeism, and increased use of sick leave [17,20].

There is evidence about the development of sleep disorders due to exposition to socially stressful situations such as wars, economic crises, or public health risk situations [21–23]. According to previous studies from SARS or Ebola epidemic, WHS are especially vulnerable to mental health problems, including sleep disturbances [24,25].

To date, little is known about the effects of the COVID-19 pandemic on the sleep quality of workers in the health sector (WHS). Regarding a recent review on the impact of the COVID-19 pandemic on mental health, health care personnel appear to be at an increased risk of stress-related psychological symptoms compared to the general population [26]. A Italian study reported that during the outbreak of COVID-19 pandemic, frontline workers had worse sleep quality than non-frontline workers [27]. These results suggest that workers in a healthcare setting have more significant psychological distress due to direct exposure to this ongoing pandemic, thus impacting their sleep quality.

Based on the clinical relevance of sleep quality on physical and mental health and the direct exposition of WHS to this public health crisis, we want to expand the existing scant literature about this topic.

In this study, we aimed to assess the sleep quality of WHS during the pandemic in Austria, comparing frontline WHS to those not directly engaged in the care of COVID-19 patients (non-frontline WHS).

We hypothesized that (1) the COVID-19 pandemic negatively influenced the sleep quality of WHS in Austria. Furthermore, we hypothesized that (2) during lockdown as well as after loosened restrictions, frontline WHS had a worse sleep quality compared to the non-frontline WHS.

## Materials and methods

### Setting and participants

An anonymized, self-reported online questionnaire survey was conducted at the Medical University of Graz, Austria, Department of Psychiatry and Psychotherapeutic Medicine, as a part of the study “Psychosocial effects of the SARS-Cov-2 pandemic on workers in the health sector in Austria “. Another research article from this survey has been recently accepted for publication [28]. All employees of the healthcare system in Austria, who could be contacted by their work councils, healthcare facilities, professional associations, and hospital administration, were invited to participate in the online survey via LimeSurvey (Version 3.27.24; https://www.limesurvey.org/de). Considering that our investigation included healthcare workers, administrative staff, housekeepers, and technical workers, we used the term "workers in the health sector (WHS)" to describe our sample. Informed consent was given online. Participation in the online survey was voluntary and anonymous. The Medical University of Graz’s ethics committee approved the study in accordance with the current Helsinki Declaration (EK number: 32 329 ex 19/20).

Inclusion criteria included voluntary participation (confirmation of the informed consent at the first page of the online survey), pursuing a health profession in Austria and business e-mail availability. Participants who terminated the questionnaire too early did not have a valid data set; thus, they were excluded from the study. The other exclusion criteria included persons who did not have an active employment relationship in the health care system in Austria and those who refused to participate after receiving the invitation. The type of facility affiliated was used as criteria to distinguish between frontline and non-frontline WHS. Frontline WHS were defined as those professionals working in organizations dedicated to the assessment, isolation, and treatment of COVID-19 infected patients. The contact with patients who tested positive for COVID-19 infection occurred either during the daily routines in the clinical care of the infected patients or as part of a medical consult within the framework of general medical care. In comparison, WHS working in other hospital departments not directly involved in COVID-19 treatment were defined as non-frontline group.

### Study1

**Fig 1:**
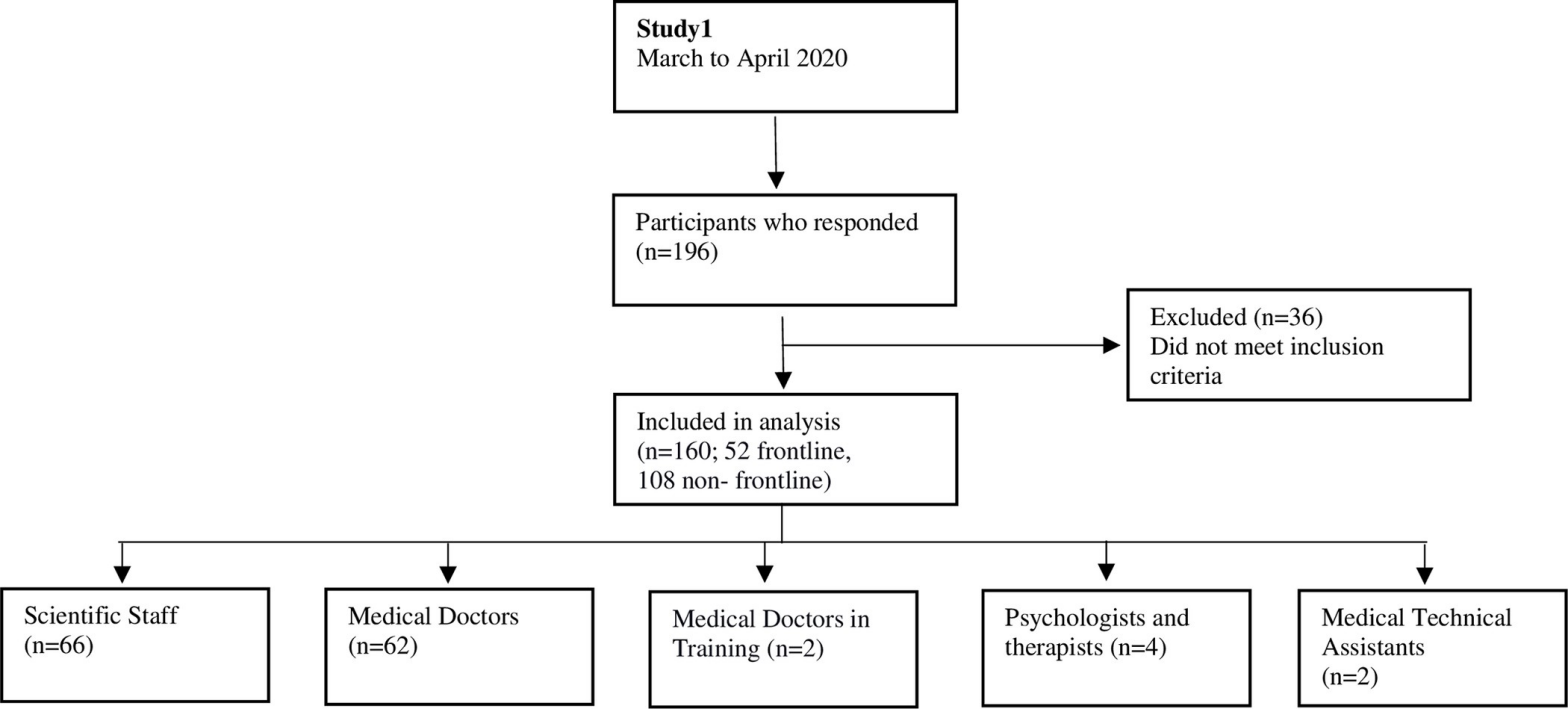
Flowchart of Study1 design and participation.

The first online survey was sent out during first strict lockdown in Austria (from March to April 2020). In the investigation were 160 participants (52 frontline, 108 non- frontline) included. Detailed information about the study procedure and the participants is graphically presented in Fig 1.

### Study2

**Fig 2:**
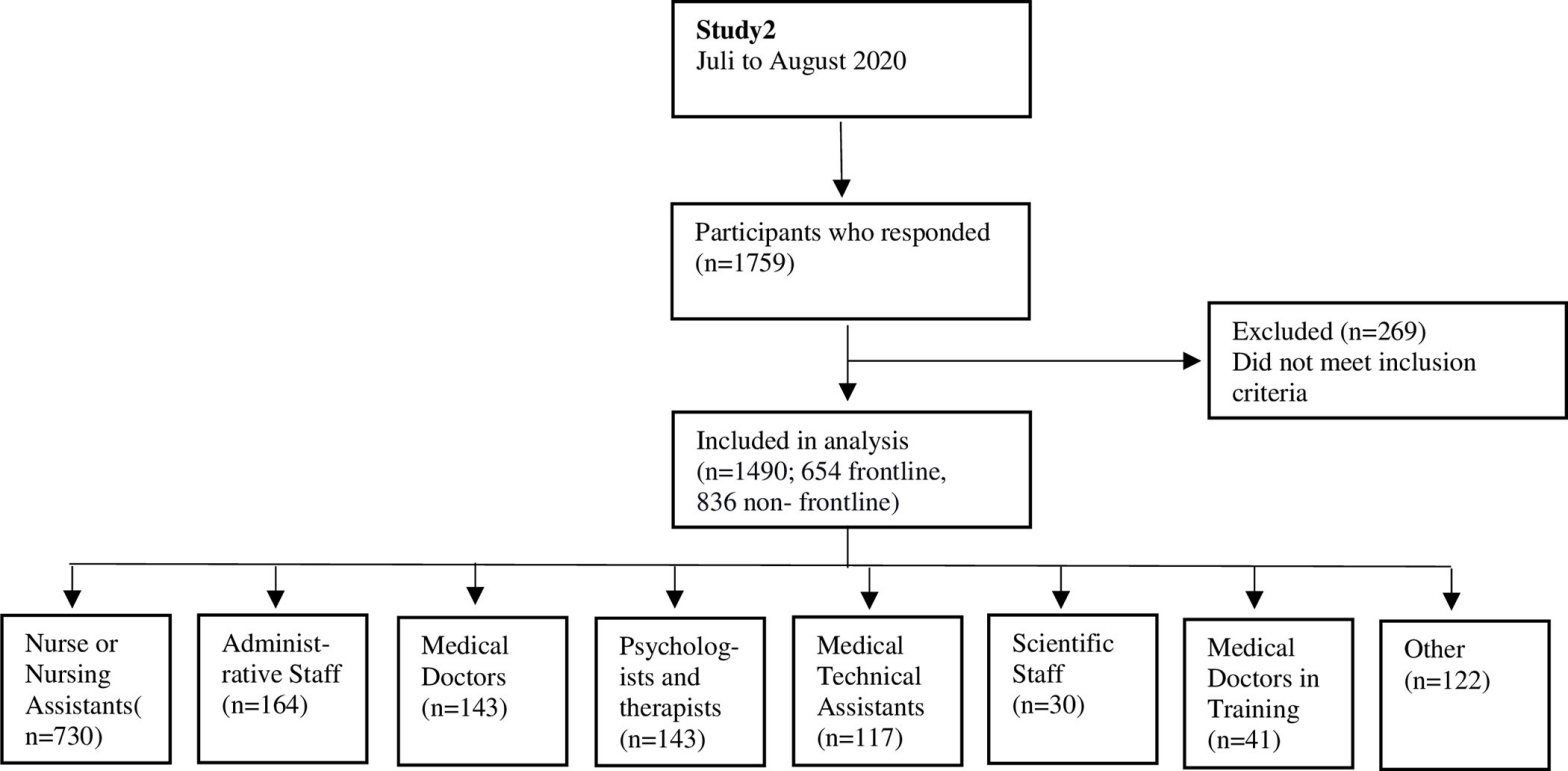
Flowchart of Study2 design and participation.

The second online survey was sent out when the social restrictions were loosened (from July to August 2021). Compared to Study1, in Study2 the number of participants and the diversity of the occupational group was larger. In total 1490 participants were included in the study, 654 of them were directly engaged in care of patients infected with COVID-19 virus (frontline workers), thus 836 belonged to the non-frontline group. A study flow diagram is presented in Fig 2.

### Materials

The current investigation was part of a large-scaled research project and thus contained several questionnaires in German language, however, only some of them were analyzed for the purpose of the current study. The socio-demographic variables, including sex, age, professional background, and type of facility affiliated with, were included in the statistical analysis. Furthermore, the self-reported questionnaire Pittsburgh Sleep Quality Index (PSQI) was used to assess the participants’ sleep quality of the previous month. The questionnaire consists of 19 items, generating seven components that produce one global score [29]. The seven components include subjective sleep quality, sleep latency, sleep duration, habitual sleep efficiency, sleep disturbances, use of sleep medication, and daytime dysfunction due to sleepiness. A total PSQI score (range 0–21) of more than five reaches a diagnostic sensitivity of 89.6% and specificity of 86.5%, whereas higher scores suggest poor sleep quality [29,30]. As the PSQI has been used in many settings, and acceptable consistency measures, internal homogeneity, and validity were obtained.

Statistics

The data were analyzed in IBM SPSS, version 26.0. We calculated the differences between frontline and non-frontline workers cross-sectionally (data from March/April and July/August separately), using Chi-Square Test (χ2), T-test and Mann-Whitney-U-Test. A two-way co-variance analysis (ANCOVA), controlled for age, was used to determine the interaction effect between the independent Study1 and Study2.

## Results

### Sleep quality of WHS in lockdown (Study1)

#### Sample description

A total of 160 of the 196 approached participants finished the survey and were included in the analysis. The largest age group were 31-40-year-olds; about 62.50% of all participants were females. Medical doctors (including medical doctors in training) represented 55.00% of the group, followed by the scientific staff with about 41.25%. Furthermore, 32.50% of WHS reported direct contact with COVID-19 patients. Table 1 provides the socio-demographic characteristics of the participants.

**Table 1:** Sociodemographic characteristics of the study participants.

	Study1				Study2			
Variable	Total (N = 160)	Frontline (N = 52)	Non-frontline (N = 108)	χ2 test	Total (N = 1490)	Frontline (N = 654)	Non-frontline (N = 836)	χ2 test
Sex				χ^2^(2) = 4.13; p = .127				χ^2^(2) = 4.82; p = .089
Male	59	23	36		262	131	131	
Female	100	28	72		1123	521	702	
Other	1	1	0		5	2	3	
Professional Background				χ^2^(4) = 64.09; p < .001				χ^2^(16) = 171.92; p < .001
Medical Doctors	62	40	22		143	83	60	
Medical Doctors in Training	26	11	15		41	26	15	
Psychologists and Therapists	4	0	4		143	40	103	
Medical Technical Assistants	2	1	1		117	52	65	
Nurse or Nursing Assistants	0				730	398	332	
Scientific Staff	66	0	66		30	3	27	
Administrative Staff	0				164	31	133	
Other	0				122	21	101	

Note: Study1- strict lockdown April/May 2020; Study2- loosened restrictions, July/Agust 2020; Other include: Housekeeper, technical workers.

#### Sleep quality

The global PSQI score for the entire sample was 4.30 (M = 4.30, SD = 2.45). Furthermore, frontline WHS scored higher than the non-frontline WHS, however, the difference was not statistically significant (T120 = -.359; p = .720). A close inspection of the individual components of PSQI showed that the sleep duration of frontline workers in lockdown was significantly shorter than that of the non-frontline group (T(106.80) = -2.77; p = .006)(Table 2).

**Table 2:** T-Test of global PSQI score and individual components of PSQI.

	Study1				Study2			
	Total	Non-frontline	Frontline	p-value	Total	Non-frontline	Frontline	p-value
Global PSQI score	4.30	4.25	4.43	.720	4.34	4.08	4.67	< .001**
Sleep quality	0.94	0.89	10.38	.200	10.37	0.98	11.04	.001**
Sleep latency	0.55	0.52	0.61	.505	0.6135	0.56	0.68	.008**
Sleep duration	0.73	0.64	0.90	.006**	0.93	0.87	10.06	< .001**
Sleep efficiency	0.27	0.26	0.28	.922	0.45	0.39	0.52	.013**
Sleep disturbance	0.98	0.99	0.98	.899	10.63	10.46	10.84	.108
Use of sleep medication	0.12	0.12	0.13	.863	0.17	0.16	0.19	.351
Daytime dysfunction	0.85	0.83	0.88	.692	0.98	0.98	0.99	.817

Note: Numbers describe mean values; p-Values calculated from the unpaired samples T-Test.

**p < .05.

**Fig 3:**
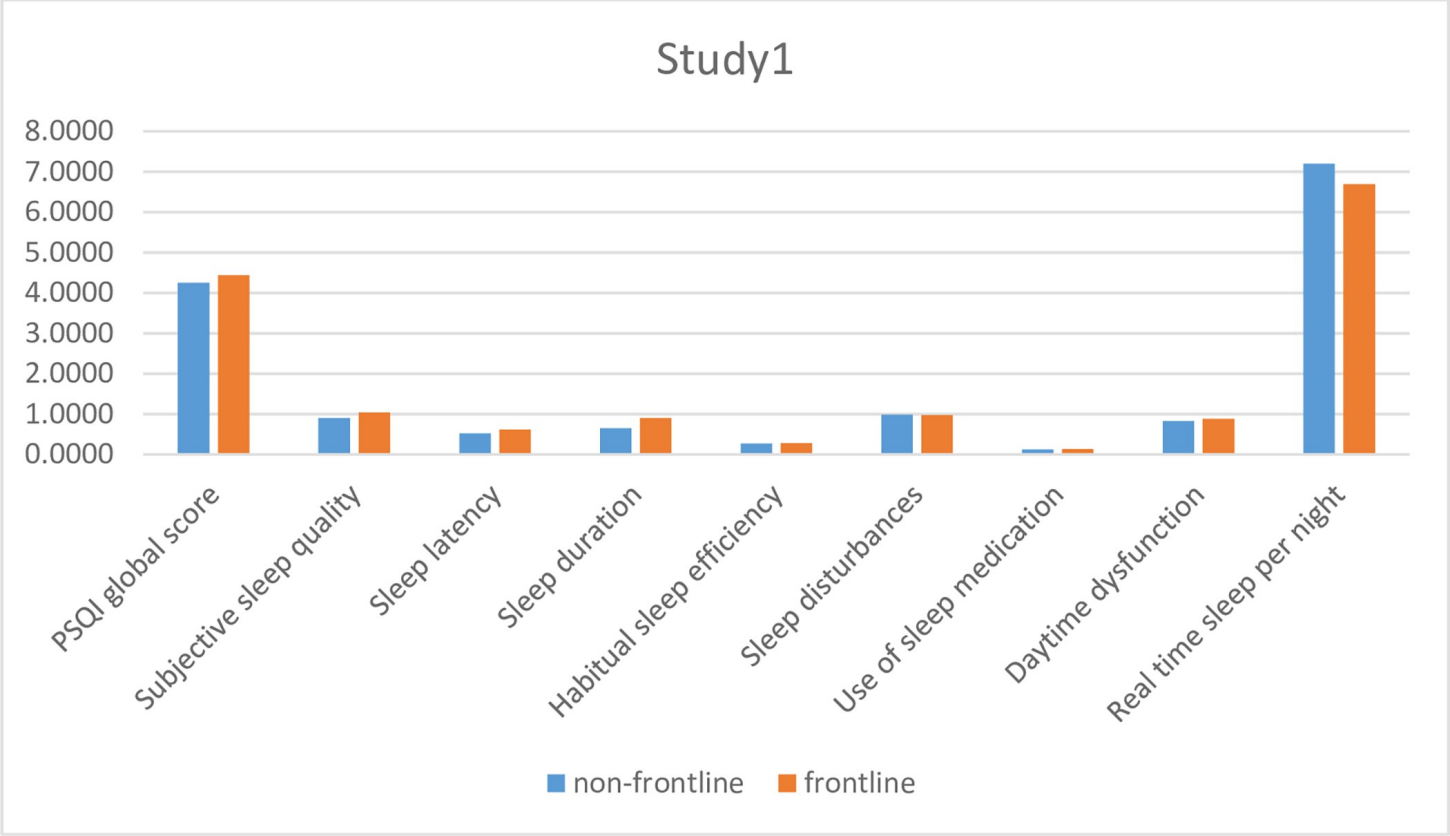
Individual components of PSQI in lockdown.

The individual components of PSQI and the differences between frontline WHS and non-frontline WHS are graphically presented in Fig 3.

### Sleep quality of WHS after loosened restrictions (Study2)

#### Sample description

From 1759 participants who initially opened the survey, 1490 completed it and were included for statistical analyses. Nursery staff represented the majority of the group with 730 participants (48.99%), followed by the medical doctors (12.34%). The largest age group in the Study2 was made up of 31-40-year-old and 41-50-year-old participants (both 19.80%), da 17.58% were represented by males and 75.36% by females. In total 43.89% of WHS were engaged in the direct care of patients positively tested for COVID-19. Table 1 provides the socio-demographic characteristics of the participants (Study2).

#### Sleep quality

**Fig 4:**
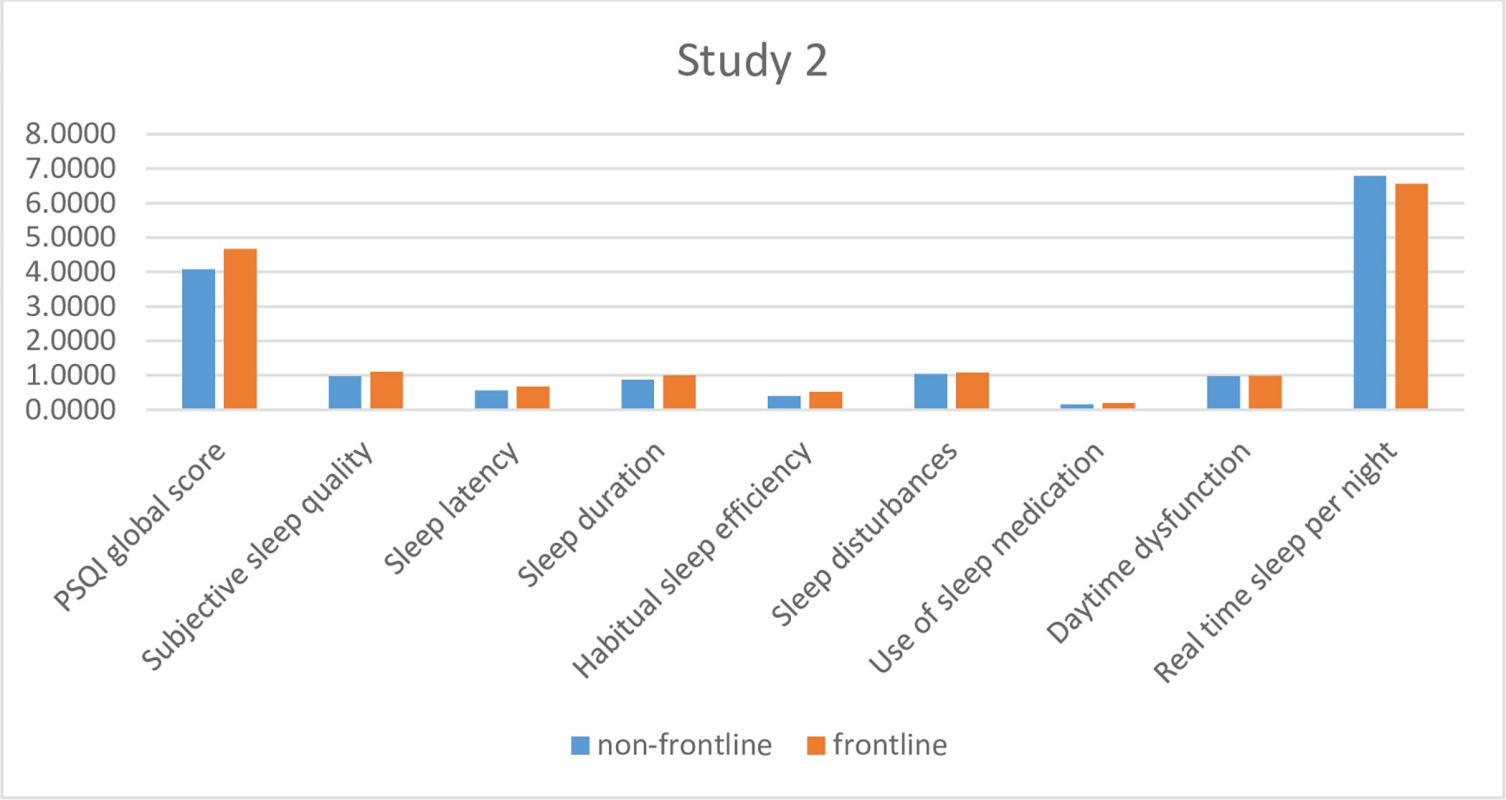
Individual components of PSQI after lossened restrictions.

The mean PSQI sum score for the entire sample in Study1 was 4.34 (M = 4.34, SD = 2.65). Furthermore, frontline WHS scored higher than the non-frontline WHS, the difference was statistically significant (T(1080) = -3.662; p < .001). A close inspection of the individual components of PSQI showed that, the subjective sleep quality (T(1252.44) = -3.194; p = .001), sleep latency (T(1174.73) = -2.667; p = .008) and sleep efficiency (T(956.53) = -2.488; p = .013) of frontline WHS was significantly worse compared to the non-frontline group (Table 2). The individual components of PSQI and the differences between frontline and non-frontline workers after loosened restrictions (Study 2) are graphically presented in Fig 4.

#### Analysis of covariance

**Fig 5:**
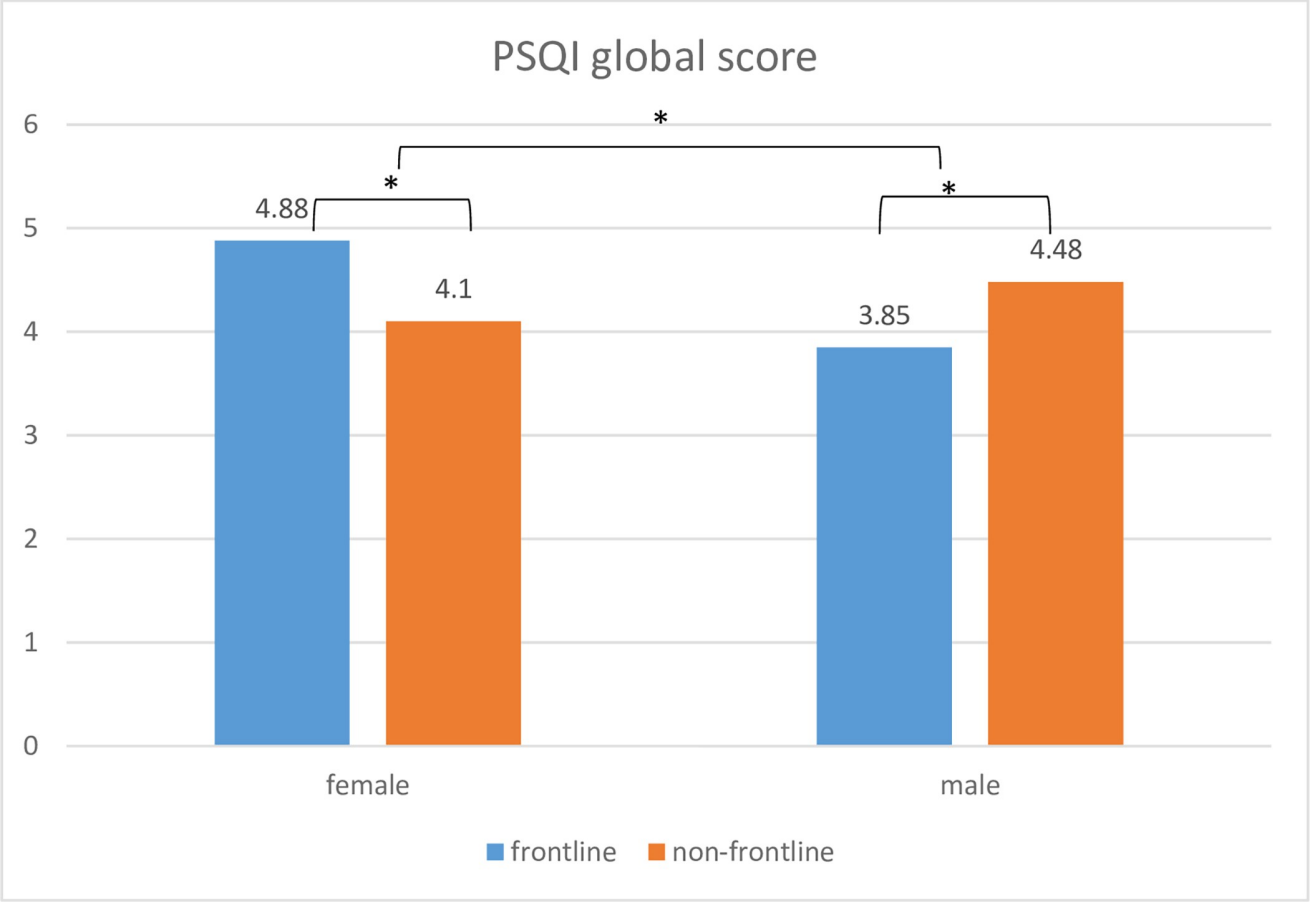
PSQI sum after loosened restrictions in frontline and non-fronline workers and both sexes.

We used two two-way ANCOVAs to analyze differences in the PSQI mean scores (controlled for age) for male vs. female between frontline vs. non-frontline WHS in both studies. In Study2, we found significant main effects for the variable sex (F (2,1070) = 3.69, p < .05) and for the variable frontline vs. non-frontline WHS (F (1,1070) = 1.38, p < .05). Women reported higher scores of PSQI than men and frontline WHS reported higher PSQI scores than non-frontline WHS (please see Fig 5). The interaction showed no significant results (p >.05).

Furthermore, the prevalence rate of poor sleep quality (PSQI >5) in Study1 was 12.30% and in Study2 was 18.20%. Frontline WHS had a higher rate of poor sleep quality in both Studies (Study1 = 18.70%, Study2 = 23.90%). Based on the professional background, we found that the medical doctors in training and nurse or nursing assistants were more likely to have a poor sleep quality than other groups of WHS. The prevalence of poor sleep in both studies is presented in Table 3.

**Table 3:** Prevalence of poor sleep quality; PSQI Cutoff > 5.

	Study1	Study2
Non-frontline	10.00%	13.70%
Frontline	18.70%	23.90%
Total	12.30%	18.20%
Professional Background		
Medical doctors	7.30%	12.60%
Medical doctors in Training	25.00%	6.50%
Psychologists and Therapists	0%	11.90%
Medical Technical Assistants	0%	9.10%
Nurse	/	20.70%
Nursing Assistants	/	33.30%
Scientific Staff	12.30%	3.60%
Administrative Staff	/	18.50%

Note: “/ “means no participation in the survey.

## Discussion

In this investigation, we focused on sleep quality of WHS during the COVID-19 pandemic in Austria. According to our first study survey, we observed that during the strict lockdown in Austria frontline WHS had a higher global PSQI score than the non-frontline WHS; however, the difference between these groups was statistically not significant. In addition, a close inspection of the individual components of PSQI showed that the sleep duration of frontline WHS in lockdown was significantly shorter than that of the non-frontline group.

In our second study we observed closely the sleep quality of WHS four months after the Austrian breakdown after a strict lockdown during times of loosened restrictions. We found a significantly worse sleep quality of frontline group than the non-frontline group. Inspecting the individual components of PSQI, we observed a significantly worse subjective sleep quality and sleep efficiency as well as a prolonged sleep latency of frontline WHS. These results are not unexpected, as other studies showed a high prevalence of poor sleep quality among healthcare workers [6,31,32]. Furthermore, female workers scored higher in the PSQI indicating a worse sleep than male workers. In addition, nurses and nursing assistants had a higher prevalence of poor sleep quality than other occupational groups. It could represent a higher amount of stress due to greater and nearer exposition to COVID-19 patients in this subgroup, as they are closer, more frequent, and more prolonged in contact with infected patients, as outlined in previous reports [33]. The prevalence rate of poor sleep quality in our investigation is in line with previous studies and emphasizes the negative impact of COVID-19 pandemic on sleep quality of WHS.

In addition, in our investigation, a direct impact of the working environment and duration of exposition was significant. The mean PSQI score of frontline workers in our second study was significantly higher than the non-frontline counterparts. Our finding is consistent with another study done in China at the pandemic peak in Wuhan in January/Feb 2020. The authors reported that frontline workers had a higher risk of developing mental health problems, including sleep problems than non-frontline workers [34]. In contrast, a previous study found no statistically significant difference in PSQI scores between frontline vs. non-frontline group [35]. The severity of the country’s affliction from the COVID-19 pandemic and the demands upon the healthcare system of the specific country at the time of the data survey could be a relevant factor affecting the results of the studies.

Furthermore, female WHS scored higher in the PSQI than male workers. Results regarding differences of PSQI scores between men and women are consistent with previous studies [36,37]. We assume that psychological and biological factors could contribute to these differences. A study by Redline and colleagues found that women consistently reported poorer sleep than men [38]. Some experts have suggested that the greater prevalence of anxiety in women could be a driving factor. Indeed, subjective sleep quality seems to deteriorate with increasing anxiety and depressive symptoms [39]. Regarding the other differences in circadian timing between the sexes, it has been hypothesized that slightly shorter circadian periods in women may cause them to be more out of circadian alignment and lead to the experience of greater insomnia [40].

Sex differences in sleep become apparent after the onset of puberty. The alterations in sleep architecture are associated with normal physiologic periods such as puberty, menstruation, pregnancy, and menopause. Sex steroids appear to influence sleep, as gonadectomy eliminates these differences [40,41]. According to another study, female sex and metabolic syndrome, were independently associated with being poor sleepers [42]. Furthermore, differences between women and men in the risk of developing sleep disorders have also been observed [43]. Research of insomnia and restless legs syndrome support a female predominance, whereas rapid eye movement sleep behavior disorder and Kleine-Levin syndrome are more common in men [43].

It is already known that women tend to provide much more negative appraisals to emergencies than men [44]. They have more caregiving responsibilities, are higher expected to balance work and houshold in every situation and tolerate multitasking, leading to a higher risk of developing stress-related symptoms [45,46]. In addition, women worldwide, but also in Austria are more often in atypical and involuntarily part-time occupation, being unable to insist on changes of working environments due to monetal factors and fear of loss of job (Gender Gesundheitsbericht 2019). All these factors could lead to a higher rate of reported sleep disturbances of women than men. However, a high amount of frontline-workers participating in our study were women which means that they were generally more exposed to close COVID-19 contact compared to men.

Notably, during the first lockdown in Austria (Study1) we found a general shorter sleep duration of frontline WHS. A recent study that evaluated the impact of lockdown due to the COVID-19 pandemic on sleep across several European countries and a South American state found that sleep duration in lockdown increased in the general population but not in health professionals [47]. The changes in the daytime routine during an epidemic, including limited working schedules and working from home, spending much more time with family members, and more time to communicate through social networks, were not applicable for health professionals in contrast to the general population. Health professionals had to work under enormous care pressure dealing with longer duty hours, work in an isolation unit, and uncertainty regarding effective disease control [6]. They had and have to work under tremendous stress of keeping themselves and their families healthy. They also have an increased risk of contracting the virus and getting sick. These factors may lead to acute sleep problems, including short sleep duration [48].

Sleep problems are commonly associated with drug and alcohol use. This association appears to be bidirectional, with sleep problems increasing the risk for developing substance use disorders and substance use leading to problems with sleep [49]. Work activity seems to impact the consumption of alcohol. Night workers are reported to have a higher propensity to consume alcohol than those who work during daytime hours, often in binge-drinking mode [50]. In a review by Cipriani and his colleagues, disordered sleep among individuals affected by dementia has been clearly demonstrated [51]. A reciprocal relationship between sleep disorders and severe medical conditions, commonly related to work and age, has also been evidenced. People with sleep disorders are usually more prone to hypertension, depression, cardiovascular and cerebrovascular diseases [48]. Nevertheless, individuals with any of these health issues are at a higher risk of developing sleep-related problems than healthy individuals. Regarding this, different work activities seem to influence the onset of diseases related to an alteration of circadian rhythm, also concerning the continuous increase of stress on the work environment associated with the recent COVID-19 pandemic that has also influenced the world of work.

The results of our study suggest that frontline WHS have been subjected to a tremendous amount of stress related to extensive exposure to new pandemic disease, directly impacting their sleep. The hospital administration should provide optimal working conditions, such as regular working hours with enough breaks, monitor the doctor-patient ratio, and offer educational and training programs to reduce stress among WHS. These preventive interventions would positively affect self-efficacy of WHS, enabling them to maintain stable emotions, promote self-control during pressure and possibly improve sleep quality.

### Limitations

This study has several limitations. To avoid possible infections, we used an online survey and self-reported tools. It is always desirable to carry out direct evaluations, although it has not been possible due to the unique circumstances of this pandemic.

Furthermore, since participation in our survey was voluntary and anonymous, it was impossible to compare the datasets, even though some subjects participated in both studies. Because of limitations from the work council departments, the online survey had to be short; therefore, we could use only a few standardized questionnaires.

## Conclusion

In conclusion, we identified a worse sleep quality in frontline WHS compared to a non-frontline group during the COVID-19 pandemic in Austria. Furthermore, female WHS scored higher in the PSQI than male WHS. In addition, nurses and nursing assistants had a higher prevalence of poor sleep quality than other occupational groups.

Preventive interventions aiming to promote good sleep quality in WHS during a healthcare crisis like this pandemic are essential to enhance resilience and mitigate the vulnerability of this specific population. Which interventions might be useful and effective has to be proven in further interventional studies in this cohort.

## Supporting information

S1 File Minimal data set- Study1. (PDF)

S2 File Minimal data set- Study2. (PDF)
