# Supplementary material for: Sleep quality among workers in the health sector exposed to the COVID-19 pandemic
Source: PLoS One. 2022 Dec 1;17(12):e0268933. doi: 10.1371/journal.pone.0268933 (PMC9714716; doi:10.1371/journal.pone.0268933)
Supplement: S1 File — (PDF) [file pone.0268933.s001.pdf]

t1

PSQI_1_SubjektiveSchlafqualität	PSQI_2rec_Schlaflatenz	PSQI_Komp_2	PSQI_3_Schlafdauer	PSQI_Komp_5	PSQI_5_Schlafstörungen	PSQI_6_Schlafmittelkonsum	PSQI_Summe_8_9	PSQI_7_Tagesschläfrigkeit	Liegezeit im Bett	Komp_4	PSQI_4_rec_Schlafeffizienz	PSQI_GES
3	2	5	1	6	1	0	1	1	10,5	57,14	3	11
2	0	1	1	11	2	0	2	1	7,5	93,33	0	6
1	0	0	0	2	1	1	1	1	8,5	94,12	0	4
2	0	1	0	4	1	0	3	2	8	100	0	5
1	2	5	0	2	1	0	3	2	9	88,89	0	6
0	0	0	1	0	0	0	0	0	6,5	92,31	0	1
1	0	0	1	2	1	0	1	1	8,25	84,85		
1	0	0	1	1	1	0	1	1	6,25	96	0	4
1	0	0	1	5	1	0	2	1	8	87,5	0	4
1	0	0	2	4	1	0	3	2	6	83,33	1	7
0	0	0	1	2	1	0	3	2	6,5	92,31	0	4
2	3	6	1	15	2	0	4	2	10,5	66,67	2	12
1	0	0	1	5	1	0	3	2	7,5	93,33	0	5
1	0	0	1	1	1	0	0	0	6,5	92,31	0	3
0	0	1	1	3	1	0	1	1	7	100	0	3
1	1	1	1	3	1	0	2	1	8,75	80	1	6
1	0	0	1	0	0	0	4	2	6	100	0	4
1	1	2	1	2	1	0	0	0	7	85,71	0	4
1	3	6	1	10	2	2	2	1	10,75	65,12	2	12
0	0	0	1	0	0	0	0	0	7,75	90,32	0	1
1	0	0	0	0	0	1	0	0	8	100	0	2
1	0	0	0	1	1	0	1	1	8	100	0	3
0	0	0	1	1	1	0	2	1	7,8	89,74	0	3
0	0	0	0	1	1	0	0	0	9	88,89	0	1
0	0	0	1	3	1	0	0	0	7	100	0	2
0	0	0	1	0	0	0	0	0	8	87,5	0	1
2	1	4	2	11	2	0	2	1	7,25	68,97	2	10
1	1	2	1	5	1	0	1	1	1	600	0	5
1	1	1	1	3	1	0	0	0	7,5	93,33	0	4
0	0	0	1	1	1	0	0	0	6,5	92,31	0	2
1	0	1	1	7	1	0	3	2	7,25	82,76	1	6
1	0	2	1	4	1	0	1	1	7,5	93,33	0	4
0	0	0	1	0	0	0	0	0	6,5	107,69	0	1
1	0	0	1	4	1	0	3	2	6,5	92,31	0	5
0	0	0	1	3	1	0	0	0	7	100	0	2
1	0	0	1	5	1	0	1	1	8	87,5	0	4
1	1	4	1	4	1	0	1	1	8	87,5	0	5
1	2	3	0	5	1	0	2	1	8,5	94,12	0	5
2	2	5	1	10	2	0	1	1	1	700	0	8
0	1	2	1	2	1	0	1	1	8	87,5	0	4
1	1	4	0	9	1	0	3	2	8,5	94,12	0	5
1	2	4	1	10	2	3	2	1	0			
0	0	0	0	2	1	0	0	0	9	100	0	1
1	3	6	0	4	1	0	3	2	10	80	1	8
1	1	3	1	5	1	0	1	1	8	87,5	0	5
1	1	3	0	8	1	0	3	2	8	100	0	5
2	1	4	1	8	1	0	2	1	7,5	93,33	0	6
1	1	1	1	10	2	0	1	1	7,5	93,33	0	6
1	0	1	1	7	1	0	0	0	19,5	35,9	3	6
1	1	2	1	0	0	0	1	1	8	87,5	0	4
1	1	2	1	9	1	0	5	3	20,5	29,27	3	10
1	0	2	1	3	1	2	2	1	7,5	93,33	0	6
0	0	0	1	0	0	0	0	0	8	87,5	0	1
1	2	5	1	6	1	0	2	1	7,75	90,32	0	6
1	0	1	0	5	1	0	1	1	8	100	0	3
1	1	3	1	0	0	0	1	1	7,42	94,34	0	4
1	1	3	1	12	2	0	1	1	8,25	84,85		
0	0	0	0	1	1	0	0	0	9,25	97,3	0	1
1	1	3	1	5	1	0	2	1	8	87,5	0	5
2	1	2	0	11	2	0	2	1				
1	1	2	0	3	1	0	0	0	9,5	84,21		

0	0	0	0	1	1	0	0	0	7,5	106,67	0	1
1	0	1	0	6	1	0	3	2	8,5	94,12	0	4
1	1	3	0	5	1	0	1	1				
1	2	5	1	2	1	0	2	1	8	87,5	0	6
1	0	0	1	4	1	0	1	1	6,5	92,31	0	4
1	1	2	0	8	1	0	2	1	13	76,92	1	5
1	1	2	0	4	1	1	1	1	9	88,89	0	5
1	1	1	1	1	1	0	0	0	7,75	90,32	0	4
1	0	1	0	4	1	0	1	1	9	88,89	0	3
1	1	2	0	9	1	0	3	2	9	88,89	0	5
0	0	1	1	4	1	0	2	1	8	75	1	4
0	0	0	0	0	0	0	0	0				
1	0	0	1	2	1	0	1	1	6	100	0	4
1	0	0	1	4	1	0	0	0	7	100	0	3
0	0	1	0	0	0	0	0	0	8	100	0	0
1	1	3	0	5	1	0	0	0	8,5	94,12	0	3
1	2	4	0	2	1	0	0	0	7,75	103,23	0	4
1	0	0	1	10	2	0	3	2	8	87,5	0	6
0	0	0	0	6	1	0	5	3	8	100	0	4
1	0	0	1	1	1	0	0	0	7	85,71	0	3
1	0	0	0	3	1	0	0	0				
1	0	1	0	3	1	0	1	1	8,75	91,43	0	3
0	0	0	1	0	0	0	0	0	6	100	0	1
1	1	2	1	4	1	0	1	1	9	77,78	1	6
3	0	0	2	8	1	0	0	0	7,25	68,97	2	8
1	0	0	0	5	1	0	2	1				
2	1	3	1	4	1	0	2	1				
1	0	1	0	4	1	0	0	0				
0	0	0	0	2	1	0	0	0	9	100	0	1
1	0	0	0	4	1	0	0	0	1	800	0	2
0	0	0	0	5	1	0	1	1	7,33	109,14	0	2
0	0	0	0	2	1	0	1	1	8	100	0	2
2	2	4	0	7	1	0	2	1	8	100	0	6
0	1	1	1	4	1	0	0	0	8	87,5	0	3
1	0	0	1	1	1	0	1	1	7,5	93,33	0	4
2	0	1	2	4	1	0	2	1				
1	0	0	0	11	2	0	0	0	8	100	0	3
1	0	0	0	1	1	0	0	0	9,5	84,21		
2	0	1	0	4	1	2	1	1	8,5	94,12	0	6
1	1	2	0	4	1	0	1	1				
0	0	0	1	1	1	0	0	0				
1	0	1	1	3	1	0	0	0				
0	0	0	0	2	1	0	0	0	9	88,89	0	1
2	0	1	2	6	1	1	3	2				
1	0	0	0	3	1	0	0	0	1	800	0	2
1	0	0	1	2	1	0	1	1	7,58	92,35	0	4
1	1	2	0	2	1	0	1	1				
0	0	0	1	1	1	0	0	0	6,75	103,7	0	2
1	1	2	0	4	1	0	1	1	9	88,89	0	4
2	2	4	1	7	1	0	3	2	8	87,5	0	8
0	0	0	1	0	0	0	0	0	7	100	0	1
2	2	5	1	5	1	0	3	2	7	85,71	0	8
1	0	1	1	3	1	0	2	1	6,5	92,31	0	4
1	1	3	1	4	1	0	3	2				
1	1	2	1	9	1	0	1	1	8,25	84,85		
0	0	0	1	0	0	0	1	1				
1	0	0	0	8	1	0	2	1	7,8	102,56	0	3
1	1	2	1	9	1	0	1	1				
1	0	1	1	1	1	0	0	0	7,75	90,32	0	3
0	0	1	1	5	1	0	0	0				
1	2	4	1	7	1	1	2	1				
1	0	0	1	5	1	0	1	1				
0	0	0	0	0	0	0	0	0	8	100	0	0
1	0	1	1	4	1	0	4	2	7,25	96,55	0	5

1	0	0	1	8	1	0	3	2	7,17	97,63	0	5
1	0	0	1	5	1	0	0	0	8	87,5	0	3
1	0	0	1	3	1	0	4	2	7	85,71	0	5
2	3	6	1	5	1	0	2	1				
1	1	2	1	5	1	0	0	0				
1	0	0	0	4	1	0	1	1	8	100	0	3
1	1	2	1	0	0	0	0	0	23,5	29,79	3	6
2	1	4	1	6	1	0	2	1	7,5	93,33	0	6
1	0	1	0	5	1	0	2	1	9	88,89	0	3
1	1	2	1	2	1	0	1	1	9	77,78	1	6
2	1	3	1	7	1	0	3	2				
1	0	0	1	12	2	0	2	1				
2	2	5	1	7	1	3	3	2				
1	1	3	1	7	1	0	0	0	8	87,5	0	4
2	1	3	1	7	1	2	2	1	8	87,5	0	8
2	0	1	2	12	2	0	2	1	8,75	57,14	3	10
0	0	1	1	6	1	0	4	2	7,5	93,33	0	4
2	1	3	0	8	1	0	0	0	8	100	0	4
1	1	4	1	8	1	0	4	2				
2	1	3	1	10	2	0	3	2	7	85,71	0	8
0	0	1	0	1	1	0	0	0	8	100	0	1
1	0	0	1	2	1	0	0	0	7	85,71	0	3
1	0	2	2	4	1	0	5	3				
0	0	0	1	1	1	0	0	0				
1	0	0	0	0	0	0	0	0	8	100	0	1
1	0	1	1	2	1	0	1	1	7,5	80	1	5
2	1	4	1	10	2	1	1	1	8,5	82,35	1	9
1	3	4	0	8	1	0	0	0				
1	1	3	1	13	2	0	0	0				
1	0	0	1	1	1	0	0	0	7	100	0	3
1	0	1	1	4	1	0	0	0	7	100	0	3
1	0	0	1	5	1	0	1	1	5,5	109,09	0	4
1	1	3	1	9	1	0	2	1				
1	1	2	1	4	1	0	1	1				
0	0	0	3	0	0	0	0	0	0			
