# Supplementary material for: Sleep quality among workers in the health sector exposed to the COVID-19 pandemic
Source: PLoS One. 2022 Dec 1;17(12):e0268933. doi: 10.1371/journal.pone.0268933 (PMC9714716; doi:10.1371/journal.pone.0268933)
Supplement: S2 File — (PDF) [file pone.0268933.s002.pdf]

t2

PSQI_1_SubjektiveSchlafqualität	PSQI_2rec_Schlaflatenz	PSQI_Komp_2	PSQI_3_Schlafdauer	PSQI_Komponente_5	PSQI_6_Schlafmittelkonsum	PSQI_Summe_8_9	PSQI_7_Tagesschläfrigkeit	PSQI_5_Schlafstörungen	PSQI_Liegezeit	Komp_4	PSQI_Komp_4_recoded	PSQI_Ges
0	0	0	3	0	0	0	0	0	7	14,29	3	3
2	0	0	1	5	0	2	1	1	7,8	76,92	1	5
0	0	0	1	2	0	0	0	1	7	100	0	1
2	1	4	1	8	1	0	0	1	8	75	1	6
2	1	4	1	10	0	0	0	2				
2	0	1	1	8	0	1	1	1	8	87,5	0	4
0	0	0	0	4	0	1	1	1				
1	0	1	1	7	0	1	1	1	7,5	93,33	0	3
0	0	0	1	3	0	1	1	1	7,8	89,74	0	2
1	0	0	0	4	0	0	0	1	1	900	0	2
0	0	0	1	3	0	0	0	1	7,5	93,33	0	1
1	0	1	0	4	0	3	2	1	8	100	0	4
									7			
2	0	0	1	6	0	1	1	1	7,5	80	1	5
0	0	0	1	1	0	0	0	1	0,3	2333,33	0	1
1	0	2	1	4	1	0	0	1	6,5	92,31	0	3
2	1	1	2	7	0	1	1	1				
0	0	1	0	6	0	3	2	1	8	100	0	3
1	0	1	0	17	0	6	3	2	1	1000	0	6
1	1	1	1	6	0	2	1	1				
0	0	1	0	3	0	0	0	1	8	100	0	1
1	1	2	1	8	0	3	2	1				
0	0	0	1	0	0	0	0	0	7	100	0	0
0	0	0	1	3	0	0	0	1	5,8	120,69	0	1
2	0	1	1	9	0	2	1	1	6,8	102,94	0	4
0	0	0	1	5	0	3	2	1				
1	1	3	0	2	0	1	1	1				
2	0	2	0	8	2	2	1	1	2,9	275,86	0	6
0	0	0	1	2	0	2	1	1	7	100	0	2
2	1	3	1	8	0	1	1	1	7,8	89,74	0	5
2	1	3	1	18	0	2	1	2	7,3	95,89	0	6
0	0	1	1	0	0	1	1	0	7,5	93,33	0	1
0	0	0	0	3	0	0	0	1				
1	1	1	1	1	0	1	1	1	6,5	92,31	0	4
1	2	4	1	3	0	1	1	1	7,5	80	1	6
1	1	2	1	2	0	0	0	1				
1	0	1	0	4	0	0	0	1	9,5	84,21		
1	0	0	0	4	1	1	1	1	1,1	727,27	0	4
1	0	1	1	6	0	1	1	1	8	87,5	0	3
1	0	1	2	1	0	0	0	1	6,2	80,65	1	3
1	0	1	0	4	0	0	0	1	8	100	0	2
2	2	5	1	5	0	1	1	1	7	100	0	6

1	2	3	1	9	0	1	1	1				
2	0	0	1	13	0	0	0	2	8	87,5	0	4
2	2	5	1	12	0	3	2	2	9	77,78	1	9
1	0	2	1	5	0	3	2	1	7	100	0	4
2	1	4	1	11	1	5	3	2	7,5	80	1	10
1	0	0	0	0	0	0	0	0	9	100	0	1
1	0	0	0	6	0	3	2	1	8	100	0	4
0	1	1	1	0	0	0	0	0	8,8	79,55	1	2
1	2	2	1	8	0	2	1	1	5,6	125	0	5
1	1	1	0	6	0	2	1	1	8,5	94,12	0	4
0	0	0	1	0	0	0	0	0	7	85,71	0	0
1	0	2	1	8	0	2	1	1				
0	0	1	0	4	0	0	0	1				
0	0	0	0	3	0	1	1	1	8,5	94,12	0	2
1	0	0	1	7	0	1	1	1	6,3	95,24	0	3
1	0	1	1	2	0	0	0	1	7,8	89,74	0	2
2	0	2	1	10	3	4	2	2	7,4	94,59	0	9
1	1	2	1	8	0	1	1	1				
1	3	4	2	8	0	0	0	1	5	100	0	5
1	1	3	2	2	0	2	1	1	7	71,43	2	6
0	1	1	1	0	0	0	0	0				
1	0	1	0	5	0	1	1	1	8,3	96,39	0	3
2	1	4	1	8	0	3	2	1	7,8	89,74	0	6
0	0	1	1	1	0	0	0	1	7	100	0	1
1	0	1	2	6	0	1	1	1	4,5	111,11	0	3
1	3	5	1	4	0	1	1	1	7,3	95,89	0	6
1	1	3	0	7	0	2	1	1	1	800	0	4
2	1	4	1	8	0	0	0	1	7,5	80	1	5
1	0	0	1	7	0	1	1	1	8	87,5	0	3
									7,5			
									7,8			
									1			
0	0	0	1	3	0	1	1	1	7,5	93,33	0	2
2	1	2	1	5	0	1	1	1				
1	0	0	1	8	0	1	1	1	8	87,5	0	3
1	0	1	0	9	0	0	0	1	8	100	0	2
1	0	1	1	2	0	0	0	1	2	300	0	2
1	0	2	1	4	0	2	1	1	8	87,5	0	3
1	0	1	1	5	0	0	0	1	6,5	107,69	0	2
2	0	0	0	6	0	5	3	1	8,8	90,91	0	6
3	3	6	2	11	0	3	2	2	5,5	90,91	0	10
1	0	0	2	5	1	4	2	1	6,8	73,53	2	7
3	3	6	3	12	1	6	3	2	6	66,67	2	14
1	1	1	0	2	0	0	0	1	9	88,89	0	3
1	1	3	0	10	0	5	3	2				
1	1	2	1	5	0	1	1	1	7	85,71	0	4
1	1	3	1	5	0	1	1	1	8,5	82,35	1	5
1	2	4	2	15	0	2	1	2	7	71,43	2	8

1	0	1	0	5	0	2	1	1	1	800	0	3
1	1	3	1	6	0	1	1	1	8,3	84,34		
1	1	3	1	4	0	1	1	1	7,8	76,92	1	5
1	1	2	1	2	0	0	0	1	6,8	88,24	0	3
0	0	0	1	0	0	0	0	0	7	100	0	0
									7,5			
1	0	0	0	2	0	1	1	1				
1	0	0	1	8	0	0	0	1				
0	0	0	1	0	0	0	0	0	7	100	0	0
1	0	1	1	3	0	1	1	1	8,5	82,35	1	4
2	1	4	1	7	3	1	1	1	6	100	0	8
1	0	0	1	3	0	0	0	1	8	87,5	0	2
1	1	2	1	8	0	1	1	1	9	77,78	1	5
									7,8			
1	2	5	1	11	0	3	2	2	8,5	82,35	1	8
1	0	0	0	1	0	0	0	1	8	100	0	2
1	0	0	1	4	0	0	0	1	7	85,71	0	2
1	1	3	0	3	0	1	1	1	1	800	0	4
1	1	2	1	4	0	1	1	1	9	77,78	1	5
1	1	1	0	2	0	1	1	1	8,8	90,91	0	4
0	0	1	0	4	0	1	1	1	8	100	0	2
0	0	0	0	1	0	0	0	1	8,5	94,12	0	1
1	0	0	0	4	0	4	2	1	9	88,89	0	4
	3	5	1	9				1				
1	0	1	1	3	0	2	1	1	7,5	80	1	4
3	2	5	3	9	0	5	3	1	7	28,57	3	12
1	0	1	0	2	0	1	1	1	7,5	106,67	0	3
1	0	0	1	3	0	2	1	1	6,5	92,31	0	3
1	0	0	1	5	0	0	0	1	7	100	0	2
1	1	2	1	6	0	2	1	1	6,5	92,31	0	4
1	2	4	1	3	0	1	1	1	9	77,78	1	6
1	0	1	0	0	0	2	1	0	9	100	0	2
1	0	0	1	7	0	0	0	1	7,5	93,33	0	2
1	3	6	1	9	0	1	1	1	9,5	73,68	2	8
0	0	0	1	0	0	0	0	0	7	100	0	0
1	0	0	1	0	1	4	2	0	8	87,5	0	4
0	0	0	1	2	0	0	0	1	7,5	93,33	0	1
1	1	1	0	5	0	2	1	1	8,5	94,12	0	4
1	0	0	1	3	0	3	2	1	7	85,71	0	4
0	0	0	1	1	0	1	1	1	7,5	93,33	0	2

1	0	0	1	5	0	0	0	1	8	87,5	0	2
1	0	2	1	3	0	1	1	1	7	100	0	3
2	1	3	1	1	0	3	2	1	7,7	77,92	1	7
0	0	0	1	1	0	2	1	1	6,5	92,31	0	2
1	1	2	0	5	1	1	1	1	6,5	123,08	0	5
1	1	1	1	2	0	0	0	1	6,5	92,31	0	3
1	0	1	2	8	1	2	1	1	9,5	52,63	3	7
1	0	2	2	7	0	1	1	1	7	71,43	2	5
2	3	3	2	8	2	2	1	1	1	500	0	9
0	0	0	1	0	0	0	0	0	0,3	2333,33	0	0
1	0	2	1	4	0	4	2	1	6,5	92,31	0	4
1	0	2	2	6	3	1	1	1				
									8			
1	1	2	1	3	0	0	0	1	1	600	0	3
1	1	2	1	8	0	3	2	1				
1	1	3	2	7	0	3	2	1	8	62,5	3	8
2	0	0	1	21	0	5	3	3	7,5	80	1	9
1	1	2	1	7	0	3	2	1	8	87,5	0	5
3	3	6	0	17	2	4	2	2	1	1000	0	12
1	0	0	1	3	0	0	0	1	7,3	95,89	0	2
1	0	1	1	3	2	1	1	1	8	75	1	6
1	0	1	1	9	0	1	1	1				
1	0	1	1	11	0	1	1	2	7	85,71	0	4
2	3	6	1	21	0	4	2	3	1,6	375	0	10
1	0	1	1	2	0	1	1	1	7,5	93,33	0	3
0	0	1	0	5	0	5	3	1	8	100	0	4
1	0	1	1	8	0	2	1	1				
2	0	0	1	5	0	2	1	1	8,5	70,59	2	6
1	1	3	1	6	0	1	1	1	7,8	89,74	0	4
1	1	3	1	5	0	2	1	1	7,3	82,19	1	5
2	2	5	1	9	0	2	1	1	6,5	92,31	0	6
1	1	3	1	7	0	4	2	1	9,5	73,68	2	7
3	0	0	2	15	0	6	3	2	7,5	66,67	2	10
2	3	6	1	11	0	6	3	2	11	63,64	3	13
1	0	0	0	8	1	0	0	1	8	100	0	3
1	1	2	1	10	0	1	1	2				
1	0	2	1	4	0	0	0	1	7	85,71	0	2
1	0	0	2	4	0	0	0	1	6,7	74,63		
2	3	6	3	8	1	4	2	1				
1	0	1	1	14	0	4	2	2	1	700	0	5
1	1	3	1	12	0	2	1	2	1	700	0	5
1	0	2	2	5	1	5	3	1	5,5	90,91	0	6
2	1	3	2	8	0	5	3	1	7,5	66,67	2	9
2	0	2	1	10	0	4	2	2	8	75	1	7

2	1	2	1	9	0	3	2	1				
0	0	0	1	0	0	0	0	0				
0	0	1	1	1	0	0	0	1	6	100	0	1
1	1	3	1	11	0	1	1	2	7	85,71	0	5
2	1	4	2	12	0	2	1	2				
2	1	3	1	1	0	3	2	1				
0	0	0	1	1	0	1	1	1	9	77,78	1	3
2	2	5	2	18	1	3	2	2	8	62,5	3	12
1	1	3	1	5	1	2	1	1	8	87,5	0	5
1	0	1	1	0	0	1	1	0	7	100	0	2
1	1	2	1	4	0	2	1	1	1	700	0	4
1	0	3	1	3	0	2	1	1	7,5	93,33	0	3
1	0	1	1	2	0	1	1	1	7	100	0	3
1	1	2	1	3	0	1	1	1	8	87,5	0	4
0	0	0	1	6	0	0	0	1	7,3	95,89	0	1
2	3	6	2	16	2	3	2	2	7	71,43	2	13
2	2	5	3	11	0	2	1	2	6,5	61,54	3	10
1	1	2	0	6	0	2	1	1	8	100	0	4
1	1	2	2	5	0	2	1	1	6,5	76,92	1	5
1	1	1	0	4	0	2	1	1	9	88,89	0	4
2	3	6	3	5	0	2	1	1	8	50	3	10
0	0	0	0	2	0	1	1	1	8	100	0	2
1	0	1	1	7	0	4	2	1	7,3	95,89	0	4
0	0	0	1	3	0	1	1	1	8,5	82,35	1	3
1	0	0	1	2	0	3	2	1	7	100	0	4
1	0	0	0	6	0	2	1	1	9	88,89	0	3
1	0	0	1	8	0	0	0	1				
									7			
1	0	0	1	3	0	0	0	1	5,8	120,69	0	2
1	1	2	1	5	0	0	0	1	6,8	102,94	0	3
1	0	0	1	4	0	2	1	1				
1	1	3	0	5	0	2	1	1				
1	0	0	0	7	0	4	2	1	2,9	275,86	0	4
1	1	2	1	5	0	1	1	1				
1	1	2	1	5	0	2	1	1	7	85,71	0	4
2	0	3	1	10	0	3	2	2	7	100	0	6
0	0	0	1	0	0	1	1	0	6	100	0	1
3	1	3	1	12	0	3	2	2	6,5	92,31	0	8

2	2	5	1	27	2	3	2	3	22,7	26,43	3	14
2	0	2	3	5	0	3	2	1	5,5	72,73	2	7
1	0	1	1	3	0	2	1	1	7	100	0	3
3	2	5	1	11	0	3	2	2	4,5	155,56	0	9
1	1	4	0	11	0	2	1	2	1	800	0	5
1	0	2	1	4	0	1	1	1	8,5	82,35	1	4
1	0	1	1	2	0	0	0	1	7,5	93,33	0	2
2	2	5	3	10	0	3	2	2	6,8	58,82	3	11
0	1	2	0	0	0	0	0	0	1	900	0	1
1	1	2	1	3	0	3	2	1	7,5	80	1	6
2	2	5	2	14	2	3	2	2	5,5	90,91	0	10
1	0	0	2	0	0	4	2	0	5,5	90,91	0	3
1	0	1	1	5	0	5	3	1	8,5	82,35	1	6
2	1	3	1	10	0	1	1	2	8,5	82,35	1	7
1	1	2	0	9	0	0	0	1				
1	2	4	1	3	0	0	0	1	1	700	0	4
1	0	0	1	3	0	1	1	1	0,5	1200	0	3
2	1	3	1	3	3	1	1	1	2,1	285,71	0	8
1	1	2	1	1	0	0	0	1	7,5	93,33	0	3
1	0	0	1	4	0	2	1	1	7,2	83,33	1	4
2	2	5	2	10	1	5	3	2	4,8	104,17	0	10
1	1	2	1	6	3	3	2	1	8	87,5	0	8
1	0	1	1	6	0	0	0	1	7,5	93,33	0	2
0	0	1	1	3	0	0	0	1	8,8	79,55	1	2
1	1	3	1	7	1	1	1	1				
1	1	3	0	2	0	1	1	1	9	88,89	0	4
0	0	0	0	3	0	1	1	1	4,1	195,12	0	2
1	1	3	0	8	0	2	1	1	9	100	0	4
0	0	0	0	0	0	0	0	0				
1	1	3	1	3	0	3	2	1	7,5	93,33	0	5
0	2	4	1	5	0	1	1	1	7,5	93,33	0	4
0	0	0	1	1	0	0	0	1	7,5	93,33	0	1
1	0	1	1	2	0	3	2	1	7	85,71	0	4
0	0	1	0	5	0	1	1	1	8,3	102,41	0	2
									2,9			
2	0	2	3	17	0	0	0	2	4,5	66,67	2	6
0	0	0	0	4	0	2	1	1	8	100	0	2
1	2	4	1	5	0	1	1	1	7	85,71	0	5

1	0	1	0	3	0	2	1	1	8	100	0	3
1	0	0	0	6	0	0	0	1	8	100	0	2
1	1	2	2	7	0	2	1	1	7	71,43	2	6
1	0	2	1	7	0	1	1	1				
1	0	2	0	4	0	2	1	1	8	100	0	3
1	1	1	1	4	0	2	1	1	8	87,5	0	4
1	0	1	0	7	2	3	2	1	9	88,89	0	6
1	0	0	1	3	0	0	0	1	7	100	0	2
0	0	0	1	5	1	1	1	1	6,3	95,24	0	3
1	0	1	0	3	0	1	1	1	9	88,89	0	3
2	1	3	0	11	0	1	1	2	7	114,29	0	6
1	0	0	1	4	0	1	1	1	7,5	93,33	0	3
2	1	4	2	17	0	2	1	2	6,5	76,92	1	7
0	1	1	1	4	0	2	1	1	8,3	84,34		
0	0	0	0	5	0	0	0	1	8,5	94,12	0	1
1	1	2	0	10	0	2	1	2	8,5	94,12	0	5
1	0	0	1	4	0	1	1	1	7,8	89,74	0	3
									7,3			
2	3	4	1	5	1	2	1	1				
1	0	0	0	8	0	1	1	1	7,5	106,67	0	3
1	0	2	1	4	0	2	1	1				
1	0	1	1	5	0	1	1	1				
1	0	0	1	8	0	1	1	1	6,5	107,69	0	3
2	1	4	1	14	1	1	1	2	7,5	93,33	0	7
1	3	3	2	0	0	0	0	0	7,3	68,49	2	6
2	2	5	1	12	0	5	3	2	6,8	88,24	0	9
0	1	1	1	2	0	0	0	1	6,8	88,24	0	2
1	0	1	1	2	0	0	0	1	6,5	92,31	0	2
1	0	1	0	12	0	5	3	2	9,5	84,21		
1	1	2	1	0	0	1	1	0	7,5	80	1	4
1	0	0	1	6	0	3	2	1	8,5	70,59	2	6
1	0	1	0	5	0	1	1	1	8	100	0	3
1	0	1	1	3	0	0	0	1	2,9	241,38	0	2
1	0	0	1	3	0	1	1	1	8,5	70,59	2	5
1	1	2	1	4	0	1	1	1	8,5	82,35	1	5
1	0	0	1	4	0	1	1	1	7,5	93,33	0	3
1	0	1	1	5	0	3	2	1	7,2	83,33	1	5
1	0	1	0	6	0	3	2	1	9,5	94,74	0	4
1	1	2	1	8	0	2	1	1	9	66,67	2	6
1	1	2	0	8	0	2	1	1	8,3	96,39	0	4
1	1	2	1	6	0	1	1	1	7,5	80	1	5
1	1	2	1	6	0	3	2	1	7,5	93,33	0	5
1	1	2	1	2	0	2	1	1	8	87,5	0	4
1	0	1	1	4	0	1	1	1	8,5	82,35	1	4
1	1	4	2	5	0	3	2	1	5	100	0	5

1	0	1	1	10	0	2	1	2	6,5	107,69	0	4
1	0	0	1	5	0	2	1	1	6,5	92,31	0	3
1	0	0	1	3	0	0	0	1	7,3	95,89	0	2
1	2	4	0	5	0	2	1	1				
1	0	2	1	11	0	3	2	2	7,5	80	1	6
1	0	0	1	6	0	3	2	1	7	85,71	0	4
2	1	2	1	6	0	0	0	1	7,5	93,33	0	4
1	2	2	0	0	0	1	1	0				
2	0	0	0	12	3	3	2	2	9,5	105,26	0	9
2	3	6	2	14	0	0	0	2	7	71,43	2	9
1	1	4	1	3	0	2	1	1	7,8	89,74	0	4
1	1	2	1	11	0	4	2	2				
1	1	1	1	2	0	0	0	1	6,5	92,31	0	3
2	2	5	2	9	3	2	1	1	6	83,33	1	10
1	0	1	1	9	0	0	0	1	4,1	146,34	0	2
0	0	0	0	1	0	0	0	1	8,5	94,12	0	1
0	0	0	1	2	0	2	1	1	6,5	92,31	0	2
1	0	0	1	6	0	1	1	1	7	85,71	0	3
1	0	1	1	7	3	1	1	1	7,3	95,89	0	6
2	1	3	1	7	0	2	1	1	7,5	80	1	6
0	1	1	1	0	0	0	0	0	6,5	92,31	0	1
2	1	2	2	7	0	1	1	1	7	71,43	2	7
1	0	0	0	1	0	1	1	1	8,5	94,12	0	3
1	1	3	1	2	0	1	1	1	8	87,5	0	4
1	2	5	1	5	2	3	2	1	8	87,5	0	8
1	0	0	1	4	0	6	3	1	7	100	0	5
									7,5			
2	2	4	3	4	0	0	0	1	7	57,14	3	8
2	2	5	1	11	0	4	2	2	6,5	92,31	0	8
1	0	0	0	0	0	2	1	0	7,8	102,56	0	2
0	0	0	1	0	0	0	0	0	6	100	0	0
1	0	0	0	4	0	1	1	1	5	160	0	3
1	0	1	0	4	0	1	1	1	9	88,89	0	3
2	2	5	1	7	3	0	0	1	9,5	63,16	3	11
1	0	0	1	3	0	0	0	1	7,3	95,89	0	2
2	0	1	1	11	3	1	1	2	7,5	80	1	9

									9,5			
									1,1			
1	1	1	1	2	0	0	0	1	8	87,5	0	3
1	1	1	1	3	0	1	1	1				
0	0	0	1	0	0	0	0	0				
1	0	0	0	6	0	0	0	1				
0	0	0	1	0	0	0	0	0				
0	0	0	1	2	0	2	1	1	8,3	84,34		
2	3	6	1	9	2	5	3	1	8,5	70,59	2	13
1	0	0	0	4	0	0	0	1	8,5	94,12	0	2
2	1	2	2	8	0	2	1	1	7,5	66,67	2	7
0	0	1	0	4	0	3	2	1	7,5	106,67	0	3
1	0	0	1	5	0	0	0	1	7,5	93,33	0	2
									9,5			
1	0	1	1	6	0	4	2	1	7,3	95,89	0	4
1	0	1	1	3	0	1	1	1	8	75	1	4
1	1	3	1	9	0	3	2	1	8	75	1	6
0	0	0	1	0	0	0	0	0	8	87,5	0	0
1	0	0	1	3	0	0	0	1	7,4	94,59	0	2
2	1	2	2	10	2	1	1	2	9	55,56	3	11
									1,1			
1	1	3	1	5	0	1	1	1	6,5	92,31	0	4
1	0	0	1	2	0	0	0	1	7	100	0	2
0	0	0	1	3	0	1	1	1	8,8	79,55	1	3
1	0	1	0	5	0	2	1	1	8	100	0	3
1	0	0	1	0	0	0	0	0	6	100	0	1
1	1	1	1	3	0	2	1	1	8,5	82,35	1	5
1	0	0	1	3	3	0	0	1	7,5	93,33	0	5
0	0	1	0	2	0	1	1	1	8	112,5	0	2
1	0	1	1	7	0	2	1	1				
1	1	3	1	5	0	0	0	1	8	87,5	0	3
1	0	0	1	3	0	2	1	1	7,3	95,89	0	3
0	0	1	1	4	0	0	0	1	7,5	93,33	0	1
3	0	0	2	18	0	5	3	2	9	55,56	3	11
0	0	0	0	0	0	0	0	0	8,5	94,12	0	0
1	0	0	2	7	0	0	0	1	6,5	76,92	1	3
0	0	0	1	2	0	0	0	1				
1	0	0	1	1	0	2	1	1	7,3	95,89	0	3
1	0	1	1	3	0	0	0	1	6,3	95,24	0	2
1	0	0	0	5	0	3	2	1	2	400	0	4
0	0	0	1	0	0	0	0	0				
1	0	0	1	4	0	0	0	1	6,5	92,31	0	2
2	1	2	1	7	0	2	1	1	6	100	0	5

1	0	0	2	2	0	1	1	1	5	100	0	3
1	1	2	0	5	0	1	1	1	8	100	0	4
1	3	6	1	17	0	1	1	2	9	66,67	2	9
1	0	0	1	5	0	0	0	1	2,7	259,26	0	2
0	0	0	0	0	0	0	0	0	7,5	106,67	0	0
2	2	5	0	12	0	6	3	2	8	100	0	9
0	0	0	1	0	0	0	0	0	7,3	95,89	0	0
1	0	2	0	0	0	2	1	0				
1	0	0	2	0	0	0	0	0	7,1	70,42	2	3
1	1	1	1	4	3	0	0	1	7,5	93,33	0	6
1	0	1	1	3	0	2	1	1	7,3	95,89	0	3
0	0	0	1	3	0	1	1	1	7,3	95,89	0	2
1	0	0	0	6	0	0	0	1	8,3	96,39	0	2
1	0	0	1	4	0	0	0	1	5,8	103,45	0	2
1	1	2	1	2	0	1	1	1	7,5	93,33	0	4
0	0	0	1	0	0	1	1	0	6,5	92,31	0	1
1	1	1	1	3	0	1	1	1				
0	0	0	1	4	0	1	1	1	6,8	102,94	0	2
1	2	5	1	8	0	2	1	1	7,5	93,33	0	5
1	0	1	1	2	0	0	0	1	7,3	95,89	0	2
1	0	1	0	7	0	1	1	1	7,8	102,56	0	3
1	2	4	1	4	1	0	0	1				
1	0	1	0	5	0	1	1	1				
1	1	1	1	0	0	2	1	0	7	100	0	3
1	0	0	1	1	0	1	1	1				
1	0	0	1	3	0	0	0	1				
1	0	1	1	1	0	2	1	1	8	87,5	0	3
2	1	3	1	4	0	3	2	1	9	77,78	1	7
									7			
2	0	0	1	10	0	2	1	2				
0	0	0	0	3	0	1	1	1	7,5	106,67	0	2
1	3	3	1	0	0	2	1	0	9	66,67	2	7
2	1	3	2	2	0	1	1	1				
1	1	3	1	5	0	1	1	1	7,5	93,33	0	4
1	0	0	2	5	0	1	1	1	7,8	64,1	3	6
0	0	1	0	4	0	0	0	1	8,3	96,39	0	1
2	1	4	1	6	0	2	1	1	6,5	107,69	0	5
1	1	2	0	10	0	4	2	2	6	150	0	6
0	0	0	0	1	0	0	0	1	9,5	94,74	0	1
0	0	0	0	1	0	1	1	1	8,5	94,12	0	2
1	0	1	0	5	0	2	1	1				
2	3	3	2	8	2	3	2	1	7,5	66,67	2	12
1	1	2	0	5	0	3	2	1				
2	3	6	2	7	0	4	2	1	8,5	58,82	3	11
1	1	2	0	3	3	1	1	1	7,8	102,56	0	7
1	0	2	1	4	0	0	0	1				

1	0	1	1	3	0	0	0	1	6,5	92,31	0	2
									8			
2	1	3	1	8	0	1	1	1	7,3	82,19	1	6
0	0	0	0	0	0	0	0	0	8	100	0	0
1	0	1	0	5	0	1	1	1	9	88,89	0	3
1	0	1	1	3	0	0	0	1	7	85,71	0	2
1	1	2	1	4	0	1	1	1	6,8	88,24	0	4
1	1	2	1	2	0	2	1	1				
2	0	0	1	6	0	0	0	1	9	77,78	1	4
0	0	0	1	0	0	0	0	0				
2	1	3	1	11	0	3	2	2	7	85,71	0	7
0	0	0	1	2	0	1	1	1	3,6	194,44	0	2
1	1	3	1	2	0	4	2	1	6,3	95,24	0	5
1	1	1	1	2	0	1	1	1	7,3	95,89	0	4
1	1	1	1	10	0	0	0	2				
1	0	0	1	14	0	3	2	2	7	85,71	0	5
1	0	1	1	3	0	0	0	1	8	87,5	0	2
2	2	4	1	8	0	2	1	1	8	75	1	7
1	0	0	1	4	0	0	0	1	4,5	155,56	0	2
0	0	0	1	2	0	0	0	1				
1	0	1	1	6	0	1	1	1	7,6	78,95	1	4
2	1	3	1	11	0	5	3	2	8	87,5	0	8
1	1	3	1	8	0	2	1	1				
0	0	1	1	5	0	0	0	1	7,5	80	1	2
2	0	2	2	3	0	1	1	1	5,3	94,34	0	4
1	0	3	1	7	1	1	1	1	8	87,5	0	4
1	0	1	0	3	0	0	0	1	7,5	106,67	0	2
2	0	1	1	12	0	3	2	2	7,8	89,74	0	6
1	0	1	0	9	0	1	1	1	8	100	0	3
1	0	0	1	7	0	2	1	1	6,5	92,31	0	3
1	0	0	0	0	0	0	0	0	7,5	106,67	0	1
									9			
1	0	0	0	0	0	1	1	0	8	100	0	2
0	0	0	1	0	0	0	0	0	8,5	82,35	1	1
0	1	1	1	1	0	0	0	1	6	100	0	2
1	0	2	0	10	0	1	1	2	8,5	105,88	0	4
2	1	3	1	14	2	4	2	2	8,5	70,59	2	11
1	1	3	1	4	1	3	2	1	6,8	88,24	0	6
3	2	5	3	20	0	4	2	3	6,5	61,54	3	13
1	0	0	1	3	0	2	1	1				

1	0	2	0	7	0	1	1	1	8,3	96,39	0	3
1	1	1	1	2	0	0	0	1	8,3	84,34		
1	0	0	1	9	0	0	0	1	8	87,5	0	2
									8			
1	0	1	0	5	0	1	1	1	8,8	90,91	0	3
0	0	1	0	1	0	1	1	1	5,6	142,86	0	2
									8,5			
1	1	1	1	0	0	2	1	0				
0	0	0	1	1	0	1	1	1				
2	1	1	1	12	0	0	0	2				
1	1	3	1	7	0	1	1	1				
1	0	0	1	9	0	1	1	1	7	100	0	3
									8,8			
1	0	1	1	8	0	2	1	1	7,5	80	1	4
1	0	0	0	4	0	0	0	1	8,3	96,39	0	2
2	0	1	0	20	0	1	1	3	6,8	117,65	0	6
0	0	1	1	1	0	0	0	1	7,3	95,89	0	1
1	0	0	1	2	0	2	1	1	5,7	105,26	0	3
1	0	0	1	0	0	2	1	0	5,5	127,27	0	2
0	1	2	0	1	0	1	1	1	9,5	84,21		
									5,6			
0	0	0	1	2	0	0	0	1				
1	2	5	2	12	0	1	1	2				
0	0	0	1	1	0	2	1	1	6,5	92,31	0	2
2	0	1	1	8	0	3	2	1	6,8	88,24	0	5
1	0	0	1	2	0	2	1	1	6,8	88,24	0	3
0	0	0	1	0	0	2	1	0	8,5	82,35	1	2
1	1	1	1	0	0	0	0	0				
1	0	0	1	3	0	1	1	1	6	100	0	3
1	2	4	0	7	0	1	1	1	8,8	90,91	0	5
0	0	0	0	1	0	1	1	1	8	100	0	2
2	0	3	1	13	0	3	2	2	8	87,5	0	6
0	0	0	1	3	0	0	0	1	2	350	0	1
1	2	5	1	8	0	2	1	1				
0	0	0	1	0	0	0	0	0	7	100	0	0
0	0	0	1	0	0	0	0	0				
0	0	0	0	2	0	0	0	1	9	100	0	1
1	0	0	0	1	0	2	1	1	1,3	615,38	0	3
1	0	1	1	1	0	0	0	1				
0	0	0	2	0	0	0	0	0	5	100	0	0

2	0	1	1	7	0	2	1	1	7	85,71	0	4
1	0	1	0	12	0	3	2	2	8,5	94,12	0	5
1	0	0	1	2	0	1	1	1	7,8	89,74	0	3
1	0	1	1	3	0	1	1	1				
1	0	0	1	2	0	1	1	1	6,5	92,31	0	3
1	0	1	1	2	0	0	0	1	7	100	0	2
1	0	0	1	3	1	4	2	1	7,3	95,89	0	5
1	0	0	1	7	0	2	1	1	6,8	102,94	0	3
1	0	0	1	3	0	0	0	1	7,5	93,33	0	2
2	0	0	2	0	0	3	2	0	7	71,43	2	6
2	0	1	1	8	0	1	1	1	5	120	0	4
2	1	3	1	4	0	1	1	1	7,3	82,19	1	6
0	0	0	1	1	0	0	0	1	6	100	0	1
2	1	3	1	4	0	6	3	1	5,7	105,26	0	7
0	0	0	0	2	0	0	0	1	8	100	0	1
1	0	2	1	7	0	1	1	1	6,8	102,94	0	3
2	2	4	1	10	0	3	2	2	7	85,71	0	8
1	0	1	0	5	0	1	1	1	8,5	94,12	0	3
1	0	1	1	4	0	1	1	1	6	116,67	0	3
2	3	6	2	15	2	4	2	2	8	62,5	3	14
1	0	1	1	3	0	1	1	1	7	100	0	3
0	1	2	1	0	0	1	1	0	7,5	93,33	0	2
2	1	3	1	4	2	2	1	1				
1	1	2	1	7	0	2	1	1				
0	0	0	1	1	0	1	1	1	7	85,71	0	2
0	0	0	1	2	0	3	2	1	6,5	92,31	0	3
1	0	2	1	2	0	0	0	1				
1	0	1	1	2	0	1	1	1	7,5	93,33	0	3
1	1	2	1	3	0	1	1	1	1,5	466,67	0	4
0	0	1	1	5	0	1	1	1	6,5	92,31	0	2
2	2	5	2	6	3	3	2	1				
1	0	0	1	6	0	3	2	1	7	85,71	0	4
1	2	2	0	3	0	1	1	1	9,2	86,96	0	5
0	1	1	0	6	0	0	0	1	9	88,89	0	2
1	0	0	1	10	0	5	3	2	6,8	88,24	0	6
1	0	3	0	5	0	2	1	1				
1	0	0	0	0	0	1	1	0	8,5	94,12	0	2
									6,3			
									7,8			
2	3	4	1	4	0	3	2	1	7,4	81,08	1	9
									5			
1	1	1	1	14	0	4	2	2	7	100	0	6
2	1	4	2	9	1	2	1	1				
									8,5			
2	2	4	2	7	0	4	2	1	7,5	66,67	2	9
1	0	0	1	3	0	1	1	1	7,5	93,33	0	3

2	3	3	3	12	2	2	1	2	5,5	72,73	2	12
1	0	3	1	7	0	0	0	1	7,3	95,89	0	2
1	2	4	3	3	0	6	3	1	5,5	72,73	2	9
1	0	0	0	1	0	0	0	1	6,3	142,86	0	2
0	0	0	0	2	0	0	0	1	8,3	96,39	0	1
1	0	2	1	4	0	3	2	1	7	85,71	0	4
1	2	4	2	4	1	1	1	1	6,7	74,63		
1	0	0	1	3	0	0	0	1	7,5	93,33	0	2
1	0	0	1	4	0	1	1	1	7,5	93,33	0	3
0	1	2	1	1	0	1	1	1	11	63,64	3	6
0	0	0	1	0	0	0	0	0	7,8	89,74	0	0
0	0	0	1	0	0	0	0	0	6,5	107,69	0	0
	0	0	0		0	0	0		7,5	106,67	0	
0	0	0	1	0	0	1	1	0	7,8	89,74	0	1
0	0	0	1	2	0	1	1	1	6,3	95,24	0	2
2	1	2	0	9	0	2	1	1	2,3	347,83	0	5
1	0	0	1	2	0	2	1	1	7,5	93,33	0	3
1	1	1	0	7	0	1	1	1	7,4	121,62	0	4
0	0	0	0	4	0	1	1	1	8,5	94,12	0	2
1	0	0	1	0	0	0	0	0	6	116,67	0	1
0	0	0	1	3	0	0	0	1	7	100	0	1
0	0	0	1	1	0	0	0	1	6,8	102,94	0	1
1	2	3	1	12	0	1	1	2	7,5	93,33	0	6
3	2	5	2	8	0	2	1	1	4	125	0	7
0	1	1	2	3	0	0	0	1	5,5	90,91	0	2
1	1	2	1	2	0	2	1	1	8	87,5	0	4
0	0	0	0	1	0	0	0	1				
2	1	3	1	6	0	1	1	1	7	85,71	0	5
2	3	6	2	11	0	3	2	2	6,5	76,92	1	10
1	0	1	1	7	2	4	2	1	7,8	89,74	0	6
2	3	5	3	5	0	3	2	1	6,4	62,5	3	11
2	0	0	1	3	0	1	1	1	7,5	80	1	5
1	0	1	1	1	0	1	1	1	7,5	80	1	4
1	0	1	0	3	0	0	0	1	9	88,89	0	2
1	0	2	1	10	0	1	1	2	5	140	0	4
1	0	2	1	6	0	1	1	1	7	85,71	0	3
1	2	2	0	6	0	4	2	1	9,3	86,02	0	6
1	2	4	1	4	0	1	1	1	8	87,5	0	5
0	0	0	1	0	0	0	0	0	7	100	0	0
1	1	3	1	4	0	1	1	1	7	85,71	0	4
1	0	1	0	4	0	1	1	1	7,8	102,56	0	3
									7			
1	0	1	0	1	0	1	1	1	8	100	0	3
0	1	1	1	6	0	1	1	1	7	100	0	3
1	0	1	1	5	0	1	1	1	6	100	0	3
1	0	1	1	3	0	4	2	1	7,3	95,89	0	4
2	1	3	1	9	2	3	2	1	6,5	92,31	0	8
1	0	0	1	2	0	1	1	1	7,5	93,33	0	3

1	0	0	1	6	0	1	1	1	8,5	70,59	2	5
1	1	1	2	2	0	0	0	1	5,5	90,91	0	3
0	0	0	1	2	0	0	0	1	6	100	0	1
1	0	0	1	3	0	1	1	1	7	85,71	0	3
1	0	0	1	8	0	5	3	1	6	100	0	5
0	0	0	0	0	0	0	0	0	9	88,89	0	0
0	0	0	1	0	0	0	0	0				
1	0	1	0	10	0	2	1	2	2,5	320	0	4
2	0	1	1	7	0	5	3	1	7,5	80	1	7
0	0	0	1	3	0	1	1	1	7,5	93,33	0	2
1	0	0	2	3	1	4	2	1				
1	1	3	1	7	0	2	1	1	7	85,71	0	4
0	0	0	1	2	0	3	2	1	8	87,5	0	3
1	0	0	1	2	0	0	0	1	7,5	93,33	0	2
1	0	0	1	4	0	0	0	1				
1	0	0	0	2	0	5	3	1	8,3	96,39	0	5
1	0	0	0	7	0	1	1	1	7,8	102,56	0	3
									7			
									4,5			
									7,3			
2	2	4	1	7	0	3	2	1	1	600	0	7
0	0	0	3	0	0	0	0	0				
									7,5			
									8,3			
1	0	0	1	2	0	2	1	1	6,5	92,31	0	3
1	0	0	0	5	1	1	1	1	8	100	0	4
0	0	0	0	2	0	3	2	1	9	88,89	0	3
1	0	1	1	3	0	1	1	1	8,8	79,55	1	4
1	1	1	1	1	0	1	1	1	7,5	93,33	0	4
0	0	0	0	3	0	0	0	1	8,3	96,39	0	1
0	0	0	1	0	0	0	0	0	7,8	89,74	0	0
0	0	0	1	2	0	0	0	1	6,5	92,31	0	1
2	0	1	0	7	0	1	1	1				
0	0	0	1	2	0	1	1	1	6,5	92,31	0	2
2	1	3	1	9	0	3	2	1	7,5	80	1	7
1	0	1	1	7	0	1	1	1	7,3	95,89	0	3
2	2	4	1	1	0	1	1	1				
2	2	4	1	7	0	1	1	1	7,5	80	1	7
2	0	2	1	8	0	2	1	1	8	87,5	0	4
1	0	0	0	2	0	2	1	1	7	128,57	0	3
1	0	0	1	2	0	0	0	1	7,3	95,89	0	2
1	0	0	1	4	0	1	1	1				
2	1	4	2	8	0	2	1	1	6,5	76,92	1	6
1	0	0	0	2	0	0	0	1	8,8	90,91	0	2
									4,5			
1	0	2	1	4	3	3	2	1	8	87,5	0	7

2	2	5	1	17	3	2	1	2	8,5	70,59	2	12
1	0	0	0	5	0	1	1	1	1	800	0	3
0	0	0	1	1	0	1	1	1	7,5	93,33	0	2
1	1	2	2	6	0	5	3	1	6,5	76,92	1	7
2	0	1	3	9	0	1	1	1	4,5	88,89	0	4
2	1	3	1	10	0	1	1	2	7	85,71	0	6
2	0	1	1	8	0	5	3	1				
1	0	0	1	5	0	0	0	1	8	87,5	0	2
1	1	1	1	2	0	0	0	1	7,8	89,74	0	3
2	0	3	2	4	0	1	1	1	7,3	68,49	2	6
1	0	1	1	3	0	4	2	1	7	85,71	0	4
1	1	3	1	2	0	0	0	1	7,3	82,19	1	4
2	3	3	3	15	0	2	1	2	4	100	0	8
1	2	4	1	2	0	2	1	1	8,5	70,59	2	7
2	2	5	2	15	3	3	2	2				
1	0	1	1	4	0	1	1	1				
0	1	1	0	13	0	1	1	2	8,4	95,24	0	4
1	0	0	1	4	0	3	2	1	7,5	93,33	0	4
1	0	0	0	12	0	4	2	2				
1	2	4	1	7	0	1	1	1	1	700	0	5
									1			
0	0	0	1	1	0	0	0	1	7	85,71	0	1
1	0	0	1	6	0	1	1	1	8	87,5	0	3
0	0	0	1	4	0	0	0	1	8,5	82,35	1	2
1	0	1	0	1	0	2	1	1	1	800	0	3
1	0	1	1	2	0	0	0	1	2,5	280	0	2
2	3	6	2	9	0	2	1	1	23	21,74	3	10
0	0	1	1	6	0	0	0	1	7,8	89,74	0	1
1	0	0	1	2	0	3	2	1	7	85,71	0	4
1	2	5	2	5	0	2	1	1	5	100	0	5
1	0	1	1	9	0	0	0	1				
2	1	2	1	10	0	1	1	2	0,3	2000	0	6
1	1	3	1	10	0	2	1	2				
2	2	4	1	4	0	1	1	1				
2	1	3	1	8	0	2	1	1	8	87,5	0	5
1	1	2	1	9	3	3	2	1	9	77,78	1	9
2	2	5	0	9	0	3	2	1				
1	1	4	1	1	0	0	0	1	8	87,5	0	3
									7,5			
2	2	5	2	7	0	3	2	1	7,5	66,67	2	9
1	0	2	0	6	0	3	2	1	9	88,89	0	4
1	2	4	2	5	0	3	2	1	6,1	81,97	1	7
0	1	2	0	9	0	0	0	1	8	100	0	2
1	0	1	1	1	0	0	0	1	6,5	92,31	0	2
2	3	5	1	10	0	2	1	2	7	85,71	0	8
1	0	1	2	2	0	3	2	1				
1	2	4	1	3	0	2	1	1				
1	0	0	0	3	0	1	1	1	8	100	0	3
	0	0	1									
1	0	0	1	8	0	3	2	1	7,5	93,33	0	4
1	1	3	1	4	0	0	0	1	7,8	89,74	0	3
0	0	1	0	4	0	1	1	1	1	800	0	2

1	2	3	1	4	0	0	0	1	7,5	80	1	5
1	2	2	2	7	0	1	1	1				
1	0	0	1	6	0	2	1	1	8	75	1	4
1	2	5	1	3	0	0	0	1				
1	1	3	2	7	0	2	1	1	8	62,5	3	7
1	0	0	1	2	0	0	0	1	2	350	0	2
									8			
									6,5			
0	0	0	1	0	0	2	1	0	8,8	68,18	2	3
3	2	5	3	18	0	6	3	2				
									5,5			
									6,8			
									6			
1	1	2	2	10	0	2	1	2	9	55,56	3	8
2	3	5	1	8	0	2	1	1				
0	0	0	1	2	0	4	2	1	7	100	0	3
									8,5			
1	1	2	1	6	3	3	2	1	7	100	0	8
1	0	0	1	8	1	0	0	1	1	600	0	3
									8,3			
2	2	5	0	13	0	3	2	2				
1	0	1	1	2	0	1	1	1	7,8	89,74	0	3
2	2	5	1	17	3	4	2	2	6,8	88,24	0	11
2	0	0	1	8	0	3	2	1	7	100	0	5
2	0	2	1	9	1	5	3	1	7,5	93,33	0	7
1	2	3	1	4	3	1	1	1				
1	1	3	1	4	0	2	1	1				
0	0	0	1	1	0	1	1	1	7	100	0	2
									8,5			
0	0	1	1	1	0	0	0	1	6	100	0	1
0	0	0	1	2	3	3	2	1				
2	2	5	1	14	0	3	2	2	8	75	1	9
1	0	1	1	6	0	2	1	1	9	66,67	2	5
1	1	1	1	2	0	1	1	1	7,8	76,92	1	5
0	0	0	1	2	0	0	0	1				
									8,5			
0	0	1	0	2	0	0	0	1				
									8			
2	0	1	3	8	0	1	1	1	7	57,14	3	7
1	0	0	0	0	0	0	0	0				
0	1	2	1	6	0	0	0	1				
1	1	2	1	6	0	2	1	1	1	600	0	4
2	2	5	1	13	0	4	2	2	9	66,67	2	10
2	2	4	1	6	1	3	2	1				
									8,8			
1	0	1	1	4	0	2	1	1	8	87,5	0	3
1	3	6	2	5	3	1	1	1				
1	0	0	1	0	0	0	0	0				
									8,5			
									9			
3	1	4	2	12	0	6	3	2				
1	0	0	1	5	0	1	1	1	7,5	80	1	4
1	0	0	1	4	0	0	0	1	7	100	0	2
1	0	2	1	5	0	1	1	1				
1	0	0	0	3	0	2	1	1				
0	0	0	1	3	0	0	0	1	7,5	93,33	0	1
1	1	2	1	1	0	4	2	1				
									6,5			
1	0	0	1	4	0	4	2	1				
1	1	3	2	7	0	4	2	1				

0	0	0	1	7	0	2	1	1				
0	0	0	1	4	0	3	2	1				
0	0	0	0	3	0	0	0	1	7	114,29	0	1
									6,5			
1	1	2	1	2	0	1	1	1	9	66,67	2	6
									9			
2	0	2	1	2	0	3	2	1	7,5	93,33	0	5
1	0	0	1	1	0	1	1	1	9,5	73,68	2	5
1	1	3	1	9	0	1	1	1				
1	0	0	0	5	0	1	1	1	7	114,29	0	3
									8			
1	0	1	1	5	0	2	1	1	7,5	80	1	4
1	1	3	1	2	0	1	1	1				
1	0	3	1	8	0	3	2	1	8,5	82,35	1	5
									7			
2	1	3	1	9	0	2	1	1	7,5	93,33	0	5
0	0	0	1	2	0	0	0	1				
1	0	0	1	4	0	1	1	1				
									8			
1	1	1	1	3	0	2	1	1				
									7			
0	0	0	1	1	0	0	0	1	7,7	90,91	0	1
1	0	0	1	2	0	1	1	1	6,5	107,69	0	3
1	0	1	1	5	0	1	1	1	6,5	107,69	0	3
2	2	5	1	14	0	4	2	2	6,5	92,31	0	8
1	0	0	1	2	0	2	1	1	9,5	73,68	2	5
1	0	0	1	7	0	3	2	1				
									8			
1	0	0	1	0	0	1	1	0	2	300	0	2
1	0	0	1	2	0	1	1	1	8	87,5	0	3
1	0	0	0	0	0	4	2	0	6,5	138,46	0	3
									8,8			
1	1	3	1	9	0	3	2	1	5,5	109,09	0	5
2	1	3	1	6	0	6	3	1	6,8	88,24	0	7
									6			
1	1	2	1	11	0	1	1	2	9	77,78	1	6
									2			
	0	0	1						6,2	96,77	0	
1	1	3	1	3	3	2	1	1	7	100	0	7
1	0	1	0	4	0	1	1	1	7	114,29	0	3
2	1	3	2	8	0	3	2	1	6,5	76,92	1	7
1	0	0	0	5	0	1	1	1	2	400	0	3
1	0	0	1	16	0	1	1	2	7,5	93,33	0	4
1	1	1	1	8	0	1	1	1	8	87,5	0	4
1	0	0	2	2	0	0	0	1	5,5	90,91	0	2
1	0	1	1	5	0	2	1	1				
0	0	0	0	0	0	0	0	0	8	112,5	0	0
1	0	2	1	5	1	3	2	1	7,3	95,89	0	5
1	1	3	1	8	0	1	1	1	8,5	70,59	2	6
0	0	0	1	0	0	0	0	0	6,2	96,77	0	0
3	1	3	1	7	0	5	3	1	7	85,71	0	8
0	0	0	0	5	0	1	1	1	6,5	123,08	0	2
1	0	0	1	1	0	1	1	1	6,5	107,69	0	3

2	2	5	1	8	1	4	2	1	6,5	92,31	0	8
0	1	1	0	2	0	0	0	1	7,5	106,67	0	2
0	1	2	1	2	0	1	1	1	7,5	93,33	0	3
1	3	5	1	11	0	3	2	2	7,3	82,19	1	9
1	0	1	1	4	0	1	1	1	8,5	82,35	1	4
2	1	3	2	6	0	3	2	1	8,8	56,82	3	9
0	0	0	0	1	0	0	0	1	2	400	0	1
1	0	1	1	5	0	1	1	1	8	87,5	0	3
1	0	1	1	8	0	1	1	1	8,9	78,65	1	4
1	0	3	1	9	0	0	0	1	7	85,71	0	2
0	0	0	1	0	0	0	0	0	6,8	102,94	0	0
1	2	5	0	2	0	2	1	1	1	800	0	5
1	1	2	1	3	0	3	2	1				
1	0	0	0	2	0	2	1	1	8	100	0	3
1	0	0	1	9	0	4	2	1	7	85,71	0	4
1	1	1	1	3	0	0	0	1	7	85,71	0	3
1	0	1	1	7	0	0	0	1	8,5	82,35	1	3
0	1	1	0	1	0	0	0	1				
1	1	1	1	0	0	0	0	0	7,8	76,92	1	3
0	1	1	1	0	0	3	2	0				
1	0	0	1	2	2	0	0	1	5,5	127,27	0	4
2	2	5	3	11	0	3	2	2	1,1	363,64	0	8
2	0	3	1	2	0	0	0	1	6	116,67	0	3
0	0	0	1	1	0	0	0	1	19,8	35,35	3	4
1	0	1	0	0	0	1	1	0	8,5	94,12	0	2
0	0	0	0	0	0	0	0	0	8	100	0	0
2	1	3	1	16	3	5	3	2	9	66,67	2	13
									6,8			
0	0	0	0	0	0	0	0	0	2	400	0	0
1	0	2	1	4	0	2	1	1	7,3	95,89	0	3
2	2	4	1	5	0	3	2	1	9	66,67	2	9
0	1	1	0	1	0	0	0	1				
1	1	1	0	5	0	1	1	1	6	133,33	0	4
1	1	3	1	4	0	1	1	1	7,5	93,33	0	4
1	0	1	1	4	0	0	0	1	7,5	93,33	0	2
1	0	0	1	2	0	0	0	1	5,5	109,09	0	2
									6			
1	0	1	1	4	0	1	1	1	8,5	70,59	2	5
1	2	5	1	5	2	2	1	1	7	85,71	0	7
2	2	5	1	15	0	4	2	2	6,3	95,24	0	8
1	0	1	1	3	0	1	1	1	7,2	83,33	1	4
1	0	0	0	2	0	0	0	1	8	100	0	2
1	0	1	1	2	0	1	1	1	9	66,67	2	5
1	0	0	0	3	0	0	0	1	8,5	94,12	0	2
2	2	3	3	4	0	0	0	1				

2	1	3	1	2	0	4	2	1	3,4	176,47	0	6
1	2	4	2	9	0	3	2	1	9	55,56	3	9
1	0	1	1	6	0	2	1	1	9,3	64,52	3	6
1	0	1	1	3	0	0	0	1	7,3	95,89	0	2
2	1	3	2	5	0	3	2	1				
2	0	1	2	13	0	4	2	2				
0	2	2	0	0	3	0	0	0	7	128,57	0	5
1	0	1	1	9	0	1	1	1	8,5	70,59	2	5
1	2	4	1	7	0	1	1	1	7	100	0	5
1	0	2	1	4	0	1	1	1	1	600	0	3
0	0	0	1	5	0	1	1	1	8,3	84,34		
									7,8			
1	1	2	1	5	0	3	2	1	6,8	88,24	0	5
									7			
									7			
1	0	0	2	3	0	1	1	1	6	83,33	1	4
0	0	0	0	3	0	0	0	1	9	88,89	0	1
2	3	6	1	8	3	4	2	1	8	75	1	12
2	0	0	1	7	0	3	2	1	7	85,71	0	5
1	0	0	1	4	0	1	1	1	7,5	93,33	0	3
									8,5			
1	1	1	0	8	0	2	1	1	9,5	94,74	0	4
1	0	1	1	6	0	3	2	1				
1	1	4	1	12	0	2	1	2	7	85,71	0	5
3	0	3	1	19	0	0	0	3	7,5	80	1	7
0	0	0	0	3	0	3	2	1				
1	1	3	1	6	0	0	0	1	8,5	82,35	1	4
1	0	0	0	1	0	1	1	1				
1	2	4	1	3	0	2	1	1	8,5	70,59	2	7
1	2	4	1	7	0	3	2	1	8,5	82,35	1	7
1	2	4	1	6	0	3	2	1	6	100	0	6
1	0	1	1	5	0	1	1	1	6,5	107,69	0	3
2	0	0	1	10	0	2	1	2	7,5	80	1	6
1	0	1	1	1	0	0	0	1	8,5	82,35	1	3
1	0	0	1	3	0	0	0	1	8,5	82,35	1	3
0	0	0	1	2	0	1	1	1				
1	1	3	1	7	0	2	1	1	9	77,78	1	5
2	0	0	2	9	0	3	2	1	6	83,33	1	6
2	0	1	1	11	1	2	1	2	1	600	0	6
1	0	1	0	6	0	1	1	1	9,5	84,21		
2	1	2	1	10	0	1	1	2	9	77,78	1	7
1	2	2	0	9	0	4	2	1	9	88,89	0	6
1	1	2	1	3	0	2	1	1	7,5	93,33	0	4
1	0	0	0	2	0	0	0	1	8,3	108,43	0	2
1	2	5	2	2	2	0	0	1	6,5	76,92	1	7
1	0	0	1	0	0	1	1	0	7	100	0	2
2	2	5	1	13	0	2	1	2	8,8	68,18	2	9
2	2	5	2	10	0	2	1	2	6	83,33	1	8
0	0	0	1	2	0	1	1	1	6,8	102,94	0	2
1	0	0	0	5	0	1	1	1	8	100	0	3

0	0	0	1	2	0	1	1	1	6	100	0	2
0	0	0	1	0	0	2	1	0				
1	0	1	1	6	0	1	1	1	5,3	113,21	0	3
1	0	0	1	4	0	2	1	1	7	85,71	0	3
1	1	4	1	7	0	5	3	1				
1	0	0	1	8	0	0	0	1	7,3	82,19	1	3
1	0	2	1	7	0	0	0	1	6,5	92,31	0	2
1	1	2	1	6	0	1	1	1	6,5	92,31	0	4
1	0	1	1	6	0	1	1	1	5,5	109,09	0	3
0	0	0	1	3	0	2	1	1	9	66,67	2	4
									7,8			
1	0	1	1	2	0	1	1	1	8,5	82,35	1	4
1	0	0	0	3	0	1	1	1	9	88,89	0	3
0	1	1	2	2	0	0	0	1	5,5	90,91	0	2
1	1	1	0	0	0	0	0	0	8	100	0	2
1	2	4	2	2	0	2	1	1	6,8	73,53	2	7
2	1	3	1	9	0	2	1	1	5,5	109,09	0	5
2	0	0	1	9	0	2	1	1	8	75	1	5
1	2	5	0	8	0	1	1	1	1	800	0	5
1	0	2	1	7	1	6	3	1	3	200	0	6
1	2	2	1	0	0	0	0	0	6,8	102,94	0	3
1	0	1	0	3	0	1	1	1	8	112,5	0	3
1	1	3	1	8	0	1	1	1	7,2	83,33	1	5
2	0	2	1	4	0	2	1	1	7	85,71	0	4
0	0	0	0	1	0	4	2	1	7	114,29	0	3
1	0	1	1	4	0	1	1	1	7,5	93,33	0	3
2	1	3	0	9	0	2	1	1	9	88,89	0	5
2	2	5	2	0	0	2	1	0	6	83,33	1	6
1	1	3	2	7	0	0	0	1	7	71,43	2	5
0	0	0	1	1	0	0	0	1	8	75	1	2
0	1	3	0	6	0	0	0	1				
1	1	2	1	4	0	2	1	1	7	85,71	0	4
1	1	3	0	7	0	2	1	1				
									7,5			
1	0	0	0	4	0	1	1	1				
1	0	0	1	4	0	2	1	1				
1	1	2	2	5	0	2	1	1				
									7			
									8,5			
									6			
0	0	0	0	0	0	0	0	0				
									8			
2	2	4	2	9	0	3	2	1	9	55,56	3	10
2	2	5	2	11	0	3	2	2	7,5	66,67	2	10
1	1	3	1	4	0	2	1	1	8	87,5	0	4
1	0	0	1	4	0	1	1	1	7,5	93,33	0	3
0	0	0	1	2	0	2	1	1	8	87,5	0	2
2	1	2	2	6	0	1	1	1	6,5	76,92	1	6
0	0	0	1	3	0	0	0	1	7,5	93,33	0	1
0	1	1	1	2	0	0	0	1				

1	1	2	0	5	0	0	0	1				
1	0	1	1	5	0	0	0	1	6,5	92,31	0	2
0	0	0	0	6	0	2	1	1	9	100	0	2
2	0	3	2	15	0	3	2	2	5,5	90,91	0	6
1	2	3	0	4	0	1	1	1	1	900	0	5
0	1	1	1	0	0	0	0	0	8	87,5	0	1
2	1	3	1	9	2	2	1	1	6	100	0	7
1	0	1	0	4	0	2	1	1				
1	0	0	1	6	0	3	2	1	7,5	80	1	5
2	0	0	1	10	0	4	2	2	7	100	0	6
1	1	2	0	10	0	4	2	2	9,5	84,21		
1	0	2	1	14	0	2	1	2	6,5	92,31	0	4
1	2	5	1	6	0	2	1	1	8	75	1	6
1	1	1	0	2	0	1	1	1				
2	1	4	1	14	0	4	2	2	8,5	82,35	1	8
0	0	1	0	3	0	1	1	1				
2	2	5	3	9	0	4	2	1	6,8	58,82	3	10
1	1	2	1	4	0	1	1	1	5,7	105,26	0	4
1	1	3	0	10	0	2	1	2	9	88,89	0	5
1	0	0	0	3	0	1	1	1	8	100	0	3
1	0	0	1	3	0	1	1	1	6	116,67	0	3
1	0	2	1	5	0	3	2	1	7,5	80	1	5
1	1	2	1	2	0	1	1	1	7,8	89,74	0	4
1	1	1	0	0	0	2	1	0	8	125	0	3
1	2	5	2	1	0	2	1	1	6,3	79,37	1	6
1	1	2	1	4	0	0	0	1				
1	0	0	1	4	0	2	1	1	7,5	93,33	0	3
1	0	1	0	2	1	1	1	1	9	88,89	0	4
1	1	2	0	0	0	1	1	0				
2	2	4	1	10	0	1	1	2	5,5	109,09	0	7
1	0	0	1	14	0	1	1	2	9	66,67	2	6
1	0	0	2	3	0	3	2	1	6,3	79,37	1	5
1	0	0	0	2	0	0	0	1	7,3 8	109,59	0	2
2	2	5	2	5	0	3	2	1	6,5	76,92	1	8

2	2	5	1	6	0	2	1	1	9	66,67	2	8
0	0	0	1	0	0	0	0	0	7,5	93,33	0	0
1	1	2	1	10	0	2	1	2	9	77,78	1	6
1	1	2	1	6	0	1	1	1	7,5	80	1	5
1	0	0	0	1	0	0	0	1	9	88,89	0	2
1	0	0	0	4	0	1	1	1	8,5	94,12	0	3
1	2	5	3	4	0	1	1	1	7	57,14	3	8
0	0	0	1	0	0	0	0	0	9	77,78	1	1
1	2	5	0	6	0	2	1	1				
1	0	0	0	7	0	0	0	1	8,5	94,12	0	2
2	2	5	2	7	0	1	1	1				
1	0	0	1	3	0	0	0	1	7	85,71	0	2
0	1	2	0	1	0	0	0	1				
1	0	2	1	5	0	3	2	1	1,1	636,36	0	4
2	1	3	2	7	1	2	1	1	7,8	64,1	3	9
1	1	2	1	5	0	1	1	1				
1	1	3	1	3	0	2	1	1	8,5	82,35	1	5
1	1	1	1	0	0	1	1	0				
2	0	0	1	5	0	1	1	1	8	75	1	5
									7			
1	2	5	0	5	0	0	0	1				
0	0	0	0	0	0	0	0	0				
1	0	2	2	7	0	3	2	1				
2	3	6	1	7	0	0	0	1				
1	0	0	0	0	0	0	0	0				
0	1	1	0	0	0	0	0	0				
2	0	1	1	6	0	3	2	1	7	100	0	5
1	0	2	1	5	0	1	1	1				
	2		3						1	400	0	
									8,5			
0	0	1	1	4	0	3	2	1	6,8	102,94	0	3
0	0	0	1	0	0	0	0	0	6,3	95,24	0	0
2	2	5	2	5	3	0	0	1				
1	0	1	1	9	0	1	1	1	7,3	82,19	1	4
0	0	0	1	2	0	1	1	1	6,5	92,31	0	2
1	1	1	0	8	0	4	2	1	9	88,89	0	5
1	0	0	1	0	0	1	1	0	4,5	133,33	0	2
1	0	1	1	6	0	2	1	1	7	100	0	3
1	1	1	1	3	0	1	1	1	8	87,5	0	4
2	0	1	1	3	0	2	1	1	8	87,5	0	4
1	0	0	1	2	3	2	1	1	7,5	93,33	0	6
1	0	0	1	0	0	2	1	0	7,5	93,33	0	2
1	0	0	1	6	0	0	0	1	7,8	89,74	0	2
1	1	3	1	6	0	0	0	1	7,5	93,33	0	3
1	2	4	2	13	1	2	1	2	8,3	60,24	3	10
2	1	4	0	6	0	0	0	1	9,8	81,63	1	5
2	2	5	1	15	1	4	2	2				
1	0	2	0	2	0	0	0	1	8,5	94,12	0	2
1	0	1	1	6	0	1	1	1	19	31,58	3	6
2	1	2	2	6	0	0	0	1	6	83,33	1	5
2	0	0	1	5	0	0	0	1	6,5	92,31	0	3

2	0	2	1	10	0	2	1	2	8,5	82,35	1	6
2	2	4	2	9	0	2	1	1	7	71,43	2	8
1	0	0	1	6	0	1	1	1	7,3	95,89	0	3
0	0	0	1	3	0	0	0	1	8	87,5	0	1
1	0	1	1	12	0	0	0	2	9	77,78	1	4
0	0	0	1	0	0	0	0	0	7	100	0	0
0	1	2	0	5	0	0	0	1	8	100	0	2
1	0	0	1	2	0	0	0	1	7	85,71	0	2
2	3	6	1	11	3	2	1	2	6	100	0	11
1	1	2	1	2	0	0	0	1	8,5	82,35	1	4
2	2	5	2	13	0	2	1	2	7	71,43	2	9
1	0	1	1	5	0	3	2	1	7,5	80	1	5
2	0	0	0	14	0	6	3	2				
1	1	2	1	6	0	0	0	1	1	700	0	3
2	2	5	1	7	0	2	1	1	8	75	1	7
0	0	0	1	3	0	3	2	1	2,6	269,23	0	3
0	0	0	0	7	0	1	1	1	9	88,89	0	2
1	0	1	0	4	0	1	1	1	1	800	0	3
1	1	2	0	7	0	1	1	1	6	133,33	0	4
0	1	1	0	5	0	0	0	1	8,5	94,12	0	2
1	0	2	1	8	0	1	1	1				
1	1	1	1	2	0	0	0	1	7,5	93,33	0	3
1	1	3	0	6	0	4	2	1	8	100	0	5
2	1	3	1	5	0	3	2	1	6,8	102,94	0	6
1	2	4	0	4	0	0	0	1				
1	0	2	1	7	0	2	1	1	6,9	101,45	0	3
1	1	4	2	23	0	3	2	3	6	83,33	1	8
2	0	1	3	6	0	5	3	1				
2	0	0	2	8	0	0	0	1	8,5	58,82	3	6
1	0	0	1	5	0	3	2	1	7,5	93,33	0	4
1	1	2	1	6	0	3	2	1	5,5	109,09	0	5
0	0	0	1	3	0	1	1	1	0,5	1200	0	2
1	1	3	1	10	3	4	2	2				
2	2	5	1	6	0	3	2	1	8,5	82,35	1	8
0	0	2	0	7	0	2	1	1	8	125	0	2
2	1	3	1	11	0	4	2	2	0,5	1200	0	7
1	0	0	1	2	0	2	1	1				
1	1	2	0	12	0	2	1	2	7	114,29	0	5
1	0	1	1	5	0	2	1	1	7,8	89,74	0	3
2	2	5	3	10	3	2	1	2	1	400	0	10
1	0	0	1	4	0	0	0	1	9	66,67	2	4
2	1	2	0	10	0	3	2	2				
									8,8			
1	0	1	0	9	0	1	1	1	8	100	0	3
1	0	0	1	7	0	4	2	1				
0	0	0	1	2	0	0	0	1				
									8,5			
1	1	2	1	4	0	0	0	1	9	66,67	2	5
1	2	3	1	7	0	0	0	1				

									9			
2	3	6	2	11	0	3	2	2	5,8	86,21	0	9
1	0	0	1	7	0	1	1	1	8	87,5	0	3
2	2	5	2	15	0	3	2	2	7,5	66,67	2	10
1	0	0	1	6	0	1	1	1	7	100	0	3
2	1	3	1	7	3	3	2	1	6,5	92,31	0	9
1	1	1	1	3	0	0	0	1				
1	1	2	0	0	0	1	1	0	9	88,89	0	3
1	0	0	1	2	0	4	2	1	7,5	93,33	0	4
1	3	5	2	5	2	1	1	1	8	62,5	3	11
1	2	4	1	12	0	4	2	2	7,5	80	1	8
3	2	5	2	9	0	3	2	1	8,5	58,82	3	11
1	1	2	1	3	0	1	1	1	6,5	92,31	0	4
0	0	1	1	6	0	2	1	1	7,8	89,74	0	2
									8,8			
0	0	0	0	0	0	2	1	0				
1	1	3	2	10	0	2	1	2	6,2	80,65	1	6
2	0	1	1	6	1	5	3	1	6,8	88,24	0	7
1	0	1	1	13	0	2	1	2	4,2	166,67	0	4
0	0	0	0	2	0	3	2	1	8	100	0	3
0	0	0	0	1	0	0	0	1	9	100	0	1
2	1	4	2	9	0	1	1	1	7,5	66,67	2	7
2	1	4	0	9	0	1	1	1				
0	0	0	0	4	0	0	0	1	8	100	0	1
2	1	3	2	6	0	0	0	1	6,5	76,92	1	5
1	0	1	1	3	0	1	1	1	7,5	93,33	0	3
1	1	2	0	4	0	0	0	1				
2	2	5	2	9	1	3	2	1	6,5	76,92	1	9
1	0	0	0	3	0	2	1	1	8	100	0	3
1	0	0	1	7	0	0	0	1	8	87,5	0	2
2	3	6	1	10	0	1	1	2				
2	0	1	2	12	0	2	1	2	23,7	21,1	3	8
0	0	0	0	0	0	0	0	0				
1	1	3	1	5	0	0	0	1	6,5	92,31	0	3
1	1	1	0	5	0	1	1	1	8	100	0	4
2	2	3	2	7	1	3	2	1	1	500	0	8
2	2	5	2	14	1	5	3	2				
1	2	4	1	8	2	2	1	1				
1	0	0	0	2	0	1	1	1	7,5	106,67	0	3
0	0	0	0	3	0	0	0	1	8	100	0	1
									8,5			
1	1	2	1	8	0	3	2	1	7,3	95,89	0	5
1	0	0	0	6	0	2	1	1	1,5	533,33	0	3

0	0	0	1	2	0	2	1	1				
1	0	0	0	1	0	1	1	1	9,5	84,21		
1	0	0	1	4	0	1	1	1	6,6	90,91	0	3
0	0	0	1	4	0	2	1	1	8,5	82,35	1	3
1	0	0	0	3	0	4	2	1				
1	0	1	1	3	0	1	1	1				
1	1	2	1	4	2	1	1	1	9	77,78	1	7
1	0	0	0	2	0	2	1	1	8	100	0	3
2	0	1	1	7	0	2	1	1				
0	1	2	0	5	0	4	2	1	1	900	0	4
1	1	3	2	4	0	4	2	1				
0	0	0	0	1	0	0	0	1				
1	0	1	0	1	0	1	1	1				
1	1	2	1	3	0	0	0	1	7	100	0	3
1	0	0	0	5	0	1	1	1	7,8	102,56	0	3
0	0	1	0	8	0	0	0	1	9	111,11	0	1
									7,5			
2	1	4	2	10	1	1	1	2	7	71,43	2	9
1	0	0	1	7	0	0	0	1				
									7,5			
0	0	0	0	0	0	0	0	0				
									6,5			
1	1	2	1	4	0	0	0	1				
1	1	3	1	13	0	2	1	2	7	100	0	5
1	1	3	1	8	0	1	1	1	7,5	80	1	5
2	0	0	2	12	0	2	1	2	6	83,33	1	6
1	0	0	1	5	0	1	1	1	6,6	106,06	0	3
1	0	1	2	3	0	1	1	1	6	83,33	1	4
1	0	0	1	14	0	2	1	2	7,2	83,33	1	5
1	1	2	1	3	0	1	1	1	8	87,5	0	4
0	0	0	1	0	0	2	1	0				
0	0	0	1	2	0	1	1	1	7,5	93,33	0	2
1	0	0	1	2	0	1	1	1	7,5	93,33	0	3
1	1	3	2	9	0	1	1	1	4,5	111,11	0	4
1	2	5	1	3	0	1	1	1	6,8	88,24	0	5
1	2	5	1	5	0	1	1	1	7	100	0	5
3	0	3	3	5	1	4	2	1				
0	0	0	1	0	0	1	1	0	7,5	93,33	0	1
1	0	1	1	6	0	0	0	1	1,5	466,67	0	2
1	1	2	1	4	0	1	1	1				
1	2	4	2	5	0	2	1	1				
0	1	1	1	6	0	4	2	1				

2	0	3	2	8	0	0	0	1				
0	1	1	1	3	0	2	1	1	6	100	0	3
2	1	2	1	4	0	1	1	1	8,3	72,29	2	7
1	0	0	1	4	0	1	1	1	7	100	0	3
									7,5			
1	1	1	1	0	0	0	0	0	1	700	0	2
0	0	1	1	4	0	0	0	1				
1	0	1	2	2	0	2	1	1	5,3	94,34	0	3
1	1	3	0	14	0	1	1	2	14	57,14	3	8
0	0	0	1	4	0	0	0	1	6,5	92,31	0	1
0	0	0	0	3	0	0	0	1	9,2	86,96	0	1
1	0	1	1	7	0	0	0	1				
1	0	1	1	7	0	3	2	1	8	75	1	5
1	0	0	1	6	0	3	2	1	6,8	88,24	0	4
2	0	2	1	14	0	2	1	2	6,5	107,69	0	5
0	1	2	1	2	0	1	1	1	6,5	92,31	0	3
1	0	0	1	3	0	0	0	1	7	100	0	2
1	1	2	0	9	0	4	2	1	1	800	0	5
1	1	3	1	6	1	2	1	1	7,2	97,22	0	5
2	0	1	1	11	0	5	3	2	8	87,5	0	7
1	1	2	3	4	0	1	1	1	4,5	88,89	0	4
1	2	5	2	15	1	1	1	2				
1	0	1	1	4	0	3	2	1	7	85,71	0	4
1	0	0	1	0	0	1	1	0	7,8	89,74	0	2
1	3	5	1	1	0	2	1	1	7,5	80	1	7
1	1	2	1	11	0	3	2	2	7	85,71	0	6
1	1	3	0	3	0	1	1	1	1	800	0	4
2	2	4	1	2	0	1	1	1	6,5	92,31	0	6
2	1	4	1	7	0	0	0	1	6,5	92,31	0	4
0	0	0	1	1	0	0	0	1				
1	0	1	1	2	0	1	1	1	7,5	80	1	4
1	1	1	1	4	0	2	1	1	6,5	92,31	0	4
1	0	1	2	5	0	1	1	1	5	100	0	3
0	0	0	0	2	0	1	1	1	9	100	0	2
1	0	1	1	1	0	2	1	1	1,8	333,33	0	3
1	1	2	1	4	0	1	1	1				
2	2	5	2	12	0	3	2	2	5,8	86,21	0	8
0	0	0	0	0	0	1	1	0				
1	0	0	1	3	0	0	0	1				
2	0	2	1	17	2	3	2	2	8	75	1	9
1	0	1	1	4	0	1	1	1	7	85,71	0	3
2	3	6	2	10	3	2	1	2	6,8	73,53	2	13

1	0	1	1	6	0	2	1	1	7,8	89,74	0	3
0	0	0	0	0	0	0	0	0	7	342,86	0	0
3	3	6	3	18	1	2	1	2	6,5	61,54	3	13
									9			
									9			
2	1	3	1	7	0	4	2	1	7,5	93,33	0	6
1	1	3	1	10	0	1	1	2	9,5	63,16	3	8
1	0	1	1	2	0	0	0	1				
1	2	5	0	12	0	3	2	2	7	114,29	0	7
0	0	0	1	2	0	1	1	1	7	100	0	2
1	0	0	1	6	0	1	1	1	8,5	82,35	1	4
1	1	2	1	2	0	0	0	1	8	87,5	0	3
0	0	0	0	5	0	2	1	1	9	100	0	2
0	0	0	0	5	0	0	0	1	9	88,89	0	1
2	1	3	1	11	0	2	1	2				
1	1	3	2	12	0	1	1	2	6,5	76,92	1	6
1	0	0	1	1	0	1	1	1	7,5	93,33	0	3
2	2	5	2	9	0	6	3	1	7	71,43	2	10
1	1	3	1	4	0	1	1	1	8,5	70,59	2	6
2	2	5	2	9	1	1	1	1	7	71,43	2	9
1	2	4	0	5	0	4	2	1	6,5	123,08	0	6
2	0	2	1	8	0	1	1	1	7,5	80	1	5
1	0	1	0	4	0	1	1	1	9	88,89	0	3
1	0	0	1	1	0	0	0	1	6,7	89,55	0	2
1	0	0	1	4	0	0	0	1	7,3	95,89	0	2
0	0	0	0	4	0	0	0	1				
1	2	5	1	5	0	2	1	1	7	100	0	5
1	0	0	0	2	0	1	1	1	1	800	0	3
0	0	0	0	0	0	0	0	0	9,5	94,74	0	0
0	0	0	0	0	0	0	0	0	8,5	94,12	0	0
1	0	0	1	0	0	1	1	0	7,5	93,33	0	2
2	2	5	1	7	0	2	1	1	9	77,78	1	7
0	0	1	1	8	0	1	1	1	9	66,67	2	4
3	2	5	2	9	0	4	2	1	7	71,43	2	10
2	1	3	2	9	0	3	2	1	7	71,43	2	8
1	1	2	0	5	0	1	1	1	8,8	90,91	0	4
2	1	4	2	8	0	2	1	1	6,5	76,92	1	6
0	0	0	1	4	0	1	1	1	8,5	82,35	1	3
2	2	4	2	14	0	2	1	2	7,5	66,67	2	9
1	0	0	0	7	0	1	1	1	9	100	0	3
1	0	0	1	2	0	0	0	1	7	85,71	0	2
2	1	4	1	10	0	2	1	2	8,5	70,59	2	8
0	0	1	1	5	0	0	0	1				
1	1	3	1	8	0	2	1	1				
1	1	3	1	6	0	2	1	1	6,5	92,31	0	4
1	0	1	1	3	0	1	1	1				
									9,5			

1	0	1	1	8	0	0	0	1	8,5	82,35	1	3
0	0	2	1	2	3	0	0	1	7,5	93,33	0	4
1	1	2	0	7	0	3	2	1	4,6	217,39	0	5
1	1	2	2	5	0	1	1	1	7	71,43	2	6
1	1	3	1	7	0	2	1	1	7,3	82,19	1	5
1	1	3	0	10	0	1	1	2	9	88,89	0	5
1	0	0	1	4	0	0	0	1				
0	0	0	1	1	0	2	1	1	6,5	107,69	0	2
1	1	4	1	7	0	2	1	1	7	85,71	0	4
1	0	0	0	14	0	0	0	2	8	100	0	3
1	1	2	0	6	0	1	1	1	1	900	0	4
1	1	3	0	9	0	4	2	1	8,5	94,12	0	5
2	1	4	2	8	1	1	1	1	9	55,56	3	9
3	2	5	3	16	0	5	3	2				
2	0	2	1	5	0	0	0	1	6,5	92,31	0	3
2	1	2	2	3	0	2	1	1	4,5	111,11	0	5
1	1	3	0	3	0	1	1	1	9	88,89	0	4
0	0	0	1	0	0	0	0	0	6,5	107,69	0	0
0	0	0	0	5	0	2	1	1	8	100	0	2
2	2	4	2	6	0	5	3	1	7	71,43	2	10
1	1	4	1	13	0	3	2	2	7	85,71	0	6
1	1	4	1	1	1	0	0	1	8,5	70,59	2	6
2	0	1	1	7	1	0	0	1	1,5	400	0	4
0	0	0	0	5	0	0	0	1	8,5	94,12	0	1
1	1	3	2	13	0	1	1	2	7,8	64,1	3	8
1	1	2	0	14	0	1	1	2	8,5	94,12	0	5
3	2	5	2	15	3	3	2	2	8	62,5	3	15
									7,5			
1	2	3	0	4	0	1	1	1				
									8,5			
									7			
									7,5			
1	1	2	1	5	0	1	1	1				
1	0	1	1	5	0	0	0	1				
									8			
1	0	0	1	4	0	0	0	1	7,5	80	1	3
0	0	0	1	2	0	0	0	1	7,5	93,33	0	1
1	1	3	1	8	0	4	2	1	7,2	97,22	0	5
0	0	0	1	3	0	4	2	1	9	77,78	1	4
0	0	1	0	2	0	2	1	1	1	800	0	2
0	1	1	0	0	0	1	1	0	1	900	0	2
1	2	4	1	11	1	2	1	2	6	100	0	7
2	1	4	0	14	0	5	3	2				
1	2	3	1	9	0	3	2	1				
0	1	1	1	1	0	1	1	1				
2	0	0	2	8	0	3	2	1	7,8	64,1	3	8
1	2	5	2	5	0	2	1	1	6	83,33	1	6

2	1	3	1	5	1	1	1	1	9	77,78	1	7
0	0	0	1	4	0	2	1	1	7,8	89,74	0	2
2	1	4	2	9	0	2	1	1	6,3	79,37	1	6
1	0	0	2	2	0	1	1	1				
1	0	0	1	2	0	1	1	1	7,3	95,89	0	3
2	2	4	1	6	2	3	2	1	6,8	88,24	0	9
0	0	0	1	3	0	1	1	1	7,4	94,59	0	2
0	0	0	1	0	0	0	0	0	6,5	92,31	0	0
1	1	1	1	2	1	0	0	1	7	100	0	4
1	0	2	1	9	0	2	1	1	6,8	102,94	0	3
1	0	0	0	1	0	0	0	1				
									8,5			
1	0	0	1	3	0	0	0	1	1,6	375	0	2
2	2	5	1	10	0	2	1	2	7	85,71	0	7
2	2	4	1	14	1	3	2	2				
2	2	4	1	3	0	1	1	1				
0	0	0	0	2	0	0	0	1	8,2	97,56	0	1
1	0	1	1	5	0	2	1	1	8,5	82,35	1	4
1	0	0	0	4	0	1	1	1	7	114,29	0	3
1	0	1	0	5	0	2	1	1	11,5	95,65	0	3
2	2	5	1	6	0	2	1	1	7,7	90,91	0	6
1	1	1	1	8	0	1	1	1	5	140	0	4
1	0	1	0	2	0	0	0	1	8,5	94,12	0	2
0	0	0	1	2	0	1	1	1	6,3	95,24	0	2
1	1	2	1	10	2	4	2	2	7,5	80	1	9
1	2	5	1	10	0	3	2	2	8,5	82,35	1	8
2	2	5	1	11	3	1	1	2	6,9	86,96	0	10
1	0	1	0	12	0	1	1	2	8,5	94,12	0	4
0	0	0	0	0	0	0	0	0	8	100	0	0
2	0	1	2	11	0	0	0	2				
2	3	6	3	9	0	4	2	1	5,6	71,43	2	10
1	1	2	1	4	0	1	1	1	9	66,67	2	6
2	0	0	1	4	0	4	2	1	7,5	93,33	0	5
1	0	0	1	6	0	1	1	1	7	100	0	3
0	1	1	2	3	0	0	0	1	9	55,56	3	5
1	1	1	3	5	0	1	1	1				
1	0	1	0	3	0	1	1	1				
1	1	4	1	9	0	2	1	1	7,6	92,11	0	4
1	0	1	1	3	0	1	1	1				
0	1	2	1	1	0	0	0	1	4,5	133,33	0	2
1	1	2	0	8	0	3	2	1	1	800	0	5

1	0	0	0	11	0	2	1	2				
0	1	1	1	5	0	0	0	1	8	87,5	0	2
0	0	0	0	3	0	0	0	1	7	114,29	0	1
1	0	0	1	4	0	2	1	1	8	87,5	0	3
0	0	0	0	3	0	1	1	1	8	100	0	2
1	0	2	1	4	0	0	0	1	7	100	0	2
1	0	0	1	7	0	3	2	1				
									7			
									7,7			
0	0	0	0	1	0	1	1	1	6,5	123,08	0	2
									6,5			
									6,5			
1	2	4	1	5	0	0	0	1	9,5	63,16	3	7
0	1	1	0	1	0	0	0	1				
3	3	6	3	22	0	2	1	3	7	57,14	3	13
1	0	1	1	8	0	1	1	1	7,2	97,22	0	3
2	2	5	1	9	0	6	3	1	5,5	109,09	0	8
2	1	2	1	11	0	1	1	2	6,5	107,69	0	6
1	0	0	1	4	0	0	0	1				
1	2	5	0	4	3	2	1	1	1	1000	0	8
3	0	0	1	3	0	3	2	1	7	85,71	0	6
1	0	0	0	7	0	2	1	1	7,7	103,9	0	3
2	1	4	2	8	0	2	1	1	8	62,5	3	8
0	0	1	0	8	0	0	0	1	8	137,5	0	1
1	1	3	1	4	0	2	1	1	1,5	400	0	4
1	0	0	2	10	0	1	1	2	6,5	76,92	1	5
1	1	2	0	3	0	1	1	1	9	111,11	0	4
1	0	2	1	9	0	4	2	1	8,5	70,59	2	6
1	1	3	0	3	0	2	1	1				
2	1	4	2	14	0	4	2	2	17,3	28,9	3	10
1	1	3	1	5	0	0	0	1				
2	0	2	1	12	0	3	2	2	3,1	193,55	0	6
1	0	0	1	4	0	1	1	1	7	100	0	3
1	0	0	1	1	0	2	1	1	7	100	0	3
1	0	1	1	10	0	2	1	2				
0	0	0	1	0	0	0	0	0				
2	0	3	2	15	0	4	2	2				
1	0	0	2	0	0	1	1	0	5,7	87,72	0	2
1	0	0	1	2	0	1	1	1				
1	0	0	1	4	0	1	1	1	5,5	109,09	0	3
1	0	1	0	2	0	1	1	1	2,5	320	0	3
1	1	2	1	4	0	1	1	1	9	77,78	1	5
0	0	0	0	0	0	0	0	0	7	114,29	0	0
1	0	1	1	5	0	1	1	1	9,5	73,68	2	5
1	0	1	1	9	0	3	2	1	6,5	92,31	0	4

1	0	0	1	3	0	0	0	1	8	75	1	3
2	1	2	2	11	0	2	1	2	6,5	76,92	1	7
0	0	0	1	3	3	1	1	1				
0	0	0	1	2	1	0	0	1	8,5	82,35	1	3
2	3	6	2	11	0	2	1	2	6,5	76,92	1	9
1	0	0	0	5	0	2	1	1	6,5	123,08	0	3
2	2	5	2	12	3	5	3	2	7	71,43	2	14
1	1	3	1	4	0	1	1	1	7,5	80	1	5
1	1	2	1	3	0	1	1	1	7,5	80	1	5
1	1	2	1	7	0	1	1	1	8,8	79,55	1	5
1	0	1	0	2	0	3	2	1	8	100	0	4
1	0	0	0	5	0	3	2	1	8,3	96,39	0	4
									6,5			
1	0	0	2	6	0	2	1	1				
0	0	0	0	3	0	0	0	1	8,8	90,91	0	1
1	2	4	1	6	0	1	1	1	6,5	92,31	0	5
1	0	1	1	6	0	0	0	1	6,8	102,94	0	2
1	0	0	1	4	0	1	1	1	8	87,5	0	3
									9,5			
1	0	0	1	5	0	1	1	1	7,8	89,74	0	3
2	0	1	2	13	0	3	2	2	5,7	87,72	0	6
1	2	4	1	11	0	4	2	2	6,3	95,24	0	7
0	0	0	1	0	0	4	2	0	8	87,5	0	2
1	2	4	1	9	0	1	1	1	8,3	84,34		
1	1	3	2	8	0	0	0	1	5	100	0	3
1	0	1	1	7	0	2	1	1	7,3	95,89	0	3
2	0	0	0	6	3	2	1	1	1	800	0	7
1	1	2	1	3	1	2	1	1	7	100	0	5
1	0	0	0	6	0	0	0	1	7,5	106,67	0	2
1	0	2	0	12	0	2	1	2	8,5	94,12	0	4
0	0	1	0	2	0	1	1	1	9	88,89	0	2
1	2	4	1	8	0	2	1	1	8,5	82,35	1	6
									7			
1	2	3	1	4	0	3	2	1	6	100	0	6
									6,5			
1	1	1	1	0	0	0	0	0	8	87,5	0	2
									1			
1	0	0	1	5	0	1	1	1	8	87,5	0	3
1	1	2	1	6	0	2	1	1	9,5	73,68	2	6
									8			
1	1	3	1	13	0	2	1	2				

2	0	2	2	13	0	2	1	2	1	500	0	5
1	2	2	1	12	0	1	1	2				
									7,3			
2	1	4	1	5	0	1	1	1	8	75	1	6
0	0	1	1	8	0	4	2	1				
2	0	1	1	7	0	3	2	1	7	85,71	0	5
1	0	1	1	5	0	1	1	1				
1	0	1	1	7	0	1	1	1	1,6	375	0	3
1	1	2	1	6	0	1	1	1	7,5	80	1	5
3	1	3	2	8	0	1	1	1	7,5	66,67	2	8
1	1	2	1	6	0	3	2	1				
									8,5			
1	0	1	1	7	0	2	1	1	7,8	89,74	0	3
2	1	3	0	10	0	4	2	2	7,3	109,59	0	7
1	1	1	1	8	0	2	1	1				
2	0	0	3	6	0	3	2	1	6,5	61,54	3	8
									9,5			
1	0	0	1	5	0	0	0	1				
									7,5			
									11			
0	0	0	0	1	0	0	0	1	8	100	0	1
1	1	3	1	3	0	1	1	1				
									7			
1	0	1	1	5	1	3	2	1	6,7	89,55	0	5
2	2	4	0	11	2	3	2	2				
2	2	4	2	11	0	4	2	2				
									1			
1	1	1	0	17	3	1	1	2				
									1			
									5,5			
1	1	3	1	8	0	1	1	1				
1	1	2	1	8	0	2	1	1	7,5	93,33	0	4
									8			
2	0	2	1	7	0	2	1	1	6	100	0	4
									7			
2	1	3	1	8	0	1	1	1				
0	0	0	1	2	0	2	1	1	9	77,78	1	3
1	0	0	1	6	0	0	0	1	8	87,5	0	2
3	0	3	3	14	0	3	2	2				
1	0	0	1	0	0	1	1	0	7,5	80	1	3
0	1	1	0	4	0	0	0	1				
1	1	2	2	6	0	5	3	1				
0	0	0	1	2	3	0	0	1				
1	0	0	0	2	0	1	1	1	1	800	0	3
1	0	0	0	3	0	0	0	1				
1	0	0	0	0	0	1	1	0				
